# Machine Learning‐Enabled Drug‐Induced Toxicity Prediction

**DOI:** 10.1002/advs.202413405

**Published:** 2025-02-03

**Authors:** Changsen Bai, Lianlian Wu, Ruijiang Li, Yang Cao, Song He, Xiaochen Bo

**Affiliations:** ^1^ Academy of Medical Engineering and Translational Medicine Tianjin University Tianjin 300072 China; ^2^ Department of Advanced & Interdisciplinary Biotechnology Academy of Military Medical Sciences Beijing 100850 China; ^3^ Tianjin Medical University Cancer Institute and Hospital Tianjin 300060 China; ^4^ Department of Environmental Medicine Academy of Military Medical Sciences Tianjin 300050 China

**Keywords:** database, deep learning, drug toxicity prediction, machine learning

## Abstract

Unexpected toxicity has become a significant obstacle to drug candidate development, accounting for 30% of drug discovery failures. Traditional toxicity assessment through animal testing is costly and time‐consuming. Big data and artificial intelligence (AI), especially machine learning (ML), are robustly contributing to innovation and progress in toxicology research. However, the optimal AI model for different types of toxicity usually varies, making it essential to conduct comparative analyses of AI methods across toxicity domains. The diverse data sources also pose challenges for researchers focusing on specific toxicity studies. In this review, 10 categories of drug‐induced toxicity is examined, summarizing the characteristics and applicable ML models, including both predictive and interpretable algorithms, striking a balance between breadth and depth. Key databases and tools used in toxicity prediction are also highlighted, including toxicology, chemical, multi‐omics, and benchmark databases, organized by their focus and function to clarify their roles in drug‐induced toxicity prediction. Finally, strategies to turn challenges into opportunities are analyzed and discussed. This review may provide researchers with a valuable reference for understanding and utilizing the available resources to bridge prediction and mechanistic insights, and further advance the application of ML in drugs‐induced toxicity prediction.

## Graphical abstract

Unexpected toxicity accounts for 30% of drug development failures. This review highlights ML innovations in predicting drug‐induced toxicity, emphasizing comparative analyses, interpretable algorithms, and multi‐source data integration. It categorizes toxicity types, summarizes ML models, and organizes key databases, offering strategies to address challenges. This work bridges prediction and mechanistic insights, advancing drug toxicity research.

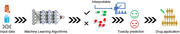

## 1 Introduction

Over the past few decades, significant progress has been made in the development of drug discovery. From traditional natural products to synthetic chemicals, and then to the latest biotechnological products, the development in the field of medicine has provided more options and opportunities for disease treatment.^[^ ^1^ ^]^ However, with the emergence of new drugs, concerns about their potential adverse reactions and toxicity have also increased. Coupled with the complexity and variety of drug toxicity, the fact that research models for one type of toxicity are often not applicable to another is a big challenge for toxicity studies. Therefore, identifying the toxicity of various drugs is essential for assessing their harmful effects on humans, animals, and the environment, which can facilitate early screening of potentially toxic chemicals and minimize the expense and risk of biological experiments.

Conventional toxicity assessments rely on cellular and animal models, which are time‐ and cost‐intensive, and raise ethical concerns. Results are often unreliable due to cross‐species differences. These methods also fail to mimic the complexity and diversity of the human body and generate a limited amount of data.^[^ ^2^ ^]^ Big data and artificial intelligence (AI), especially machine learning (ML), are powerfully advancing innovation and progress in toxicity assessments.^[^ ^3^ ^]^ ML offers faster toxicity assessments than animal studies and helps reduce reliance on animal testing.^[^ ^4^ ^]^ Furthermore, some studies have proven that ML methods trained on big data can be comparable or even superior to expensive animal experiments in discriminating certain toxicities.^[^ ^5^ ^]^ While limited training data remains a challenge in toxicity studies.^[^ ^6^ ^]^ Integrating multiple data sources can improve the accuracy and interpretability of models.^[^ ^7^ ^]^ Some studies summarized the application of ML in drug toxicity prediction from certain perspectives of computational models or molecular representation.^[^ ^4^, ^8^ ^]^ However, comprehensive databases and tools, and interpretable algorithmic models for prediction of different types of toxicity are not well elucidated in existing research.

**Figure 1:**
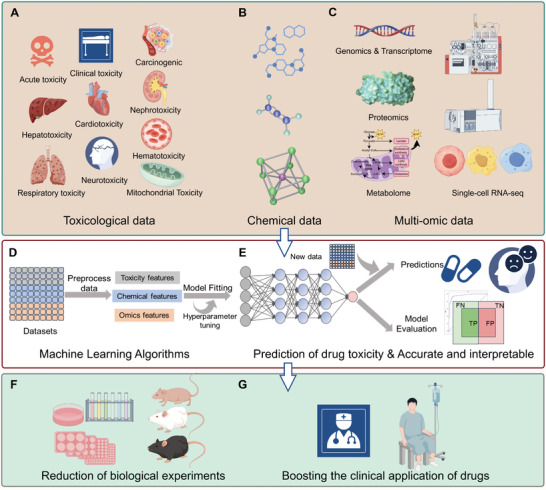
The comprehensive framework for drug toxicity prediction using multimodal data and ML: A data‐driven approach to reduce biological experimentation and enhance drug safety. The first part (Figures 1A–C) shows the integration of various data sources‐toxicity data, chemical data, and multi‐omics information (including genomics, proteomics, metabolomics, and single‐cell sequencing) into an ML pipeline designed for accurate drug toxicity prediction. The second part (Figures 1D, E) details the stepwise process of data preprocessing, model fitting, prediction, and evaluation, including preprocessing raw data into relevant features such as chemical properties, toxicity markers, and omics data, followed by model fitting and hyperparameter tuning. The performance of models is evaluated using standard metrics (True Positive, TP; True Negative, TN; False Positive, FP; False Negative, FN) to ensure prediction accuracy and interpretability. The third part (Figures 1F, G) compares traditional toxicity testing methods with this data‐driven ML approach, significantly reducing the need for in vivo biological testing. It highlights how this integrated method can improve the clinical application of drugs and decrease the demand for extensive biological experiments. This framework provides a more effective pathway for identifying potential drug toxicity risks before clinical trials, facilitating faster and safer drug development.

In this study, we reviewed drug‐induced toxicity prediction studies published over the past five years, providing a comprehensive and extensive analysis of toxicity prediction trends, labels, and representation databases for drugs (**Figure** **1**). Drugs discussed in this review include small‐molecule drugs and drug candidates (small‐molecule chemicals, peptides, natural products, etc.). By grouping toxicities based on biological characteristics and mechanisms—such as acute effects, organ‐specific impacts, or molecular‐level disruptions—we provide a clearer understanding of how toxic effects manifest in vivo. Our contributions to this review include:

1. A categorization of 10 complex drug‐induced toxicities, summarizing the characteristics of the toxicity and the applicable ML models. We compare and analyze the prediction models and interpretable methods applied in each toxicity field for multiple complex toxicities, bridging prediction and mechanistic insights, to strike a balance between breadth and depth.
2. Summarize the datasets and tools used in the field of drug toxicity prediction, including 55 databases and 12 tools. We categorized the databases into four main groups: toxicity databases, chemical databases, omics databases, and benchmark databases. We consolidated the databases according to their main areas of interest and unique features and presented the databases in subcategories under each major category to clearly outline their contribution to drug toxicity prediction.
3. Challenges and opportunities for drug toxicity prediction using ML are summarized. We analyze and discuss how challenges can be turned into opportunities to further advance the development of drug toxicity prediction.

## 2 Applications of ML in Drug Toxicity Prediction

Here, drug‐related toxicities suitable for predictive modeling were categorized into four major groups and 10 specific types: acute and clinical toxicity, carcinogenicity, organ‐specific toxicity (hepatotoxicity, cardiotoxicity, nephrotoxicity, respiratory toxicity, neurotoxicity), and cellular/molecular toxicity (hematotoxicity, mitochondrial toxicity). The following sections will compare and discuss existing prediction studies for each of these 10 toxicity types.

In drug‐induced toxicity prediction tasks, the selection of molecular representations, predictive models, and interpretability methods will significantly impact the prediction performance. Molecular representations describe the characteristics of drugs and serve as inputs for predictive models. These include molecular fingerprints (Morgan, MACCS, RDKit), molecular descriptors, and molecular graphs. Molecular fingerprints and molecular graphs focus on describing the chemical structures, while molecular descriptors are more concerned with the physicochemical properties.

**Figure 2:**
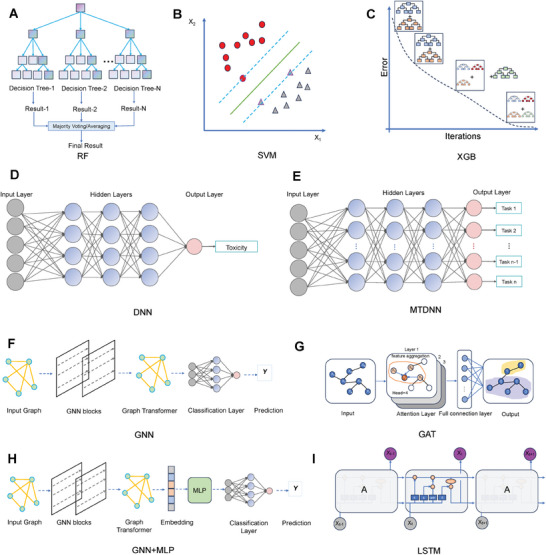
ML and DL Algorithms with Good Performance in Drug Toxicity Prediction. A) RF: An ensemble learning method that constructs multiple Decision Trees (DT) and combines their predictions to improve model accuracy. B) SVM: Maps chemical features to a high‐dimensional space and creates an optimal hyperplane boundary for classification tasks. C) XGB: An optimized implementation of gradient‐boosted decision trees (GBDT), which combines multiple weak learners (e.g., DT) to form a strong model, particularly suitable for handling complex nonlinear relationships. D) DNN: A DL model composed of multiple layers of fully connected neurons, capable of learning complex nonlinear relationships. E) Multi‐Task Deep Neural Network (MTDNN): An extended DNN that supports multi‐task learning, enabling the simultaneous prediction of multiple toxicity endpoints while sharing underlying features and task‐specific layers. F) GNN: A DL model specifically designed to handle graph‐structured data (e.g., molecular graphs). It learns the relationships between nodes (atoms) and edges (bonds) in the molecular structure and captures molecular features through a message‐passing mechanism. G) GAT: A variant of GNN that integrates an attention mechanism to assign different weights to nodes and edges in graph‐structured data, enhancing the model's ability to focus on important molecular fragments. H) GNN and Multilayer Perceptron (MLP) Joint Application: Uses graph embeddings extracted by GNN from molecular graphs as inputs, which are then passed to an MLP for further predictions. I) Long Short‐Term Memory (LSTM): An improved version of recurrent neural RNN, focused on handling sequential data and capable of capturing long‐term dependencies.

ML‐based toxicity predictive models are usually categorized into traditional ML and emerging deep learning (DL) algorithms. Traditional ML algorithms include Logistic Regression (LR), Random Forest (RF), Support Vector Machine (SVM), and eXtreme Gradient Boosting (XGB). Deep Neural Networks (DNN), Graph Neural Networks (GNN), Recurrent Neural Networks (RNN), Graph Attention Network (GAT), and Transformer are DL models applied in toxicity prediction. **Figure** **2** illustrates representative ML and DL algorithms used in drug toxicity prediction. The size of the dataset significantly influences the selection of predictive models. DL models perform best with large training datasets. However, they often achieve suboptimal performance compared to traditional ML models when trained on small toxicity datasets, as DL models typically require large amounts of data for effective training.

In drug toxicity prediction, interpretable methods are crucial for understanding and optimizing the prediction process. They help scientists interpret model behavior, identify potential toxicity mechanisms, and enhance the reliability of predictions. Interpretability methods can be categorized based on their form of output: intrinsic and post‐hoc. Intrinsic interpretability refers to models that can be intuitively explained, such as linear and LR, which directly show the weights of chemical descriptors in toxicity predictions. Post‐hoc interpretability involves external methods used to explain black‐box models. For example, SHapley Additive exPlanations (SHAP) analysis can post‐process the outputs of toxicity prediction models, identifying key molecular features affecting predictions.^[^ ^9^ ^]^ Counterfactual analysis, through generating non‐toxic analogs close to the prediction results, assists in designing molecules with lower toxicity.^[^ ^10^ ^]^

Next, we summarize the definitions of the 10 toxicity prediction tasks, the databases used, the prediction and interpretable methods, and the research advances in performance.

### 2.1 Advances in Acute and Clinical Toxicity Prediction

Acute and clinical toxicity describes the harmful effects of drugs on multiple tissues of the body or animals under different conditions and time frames. Acute toxicity focuses on the immediate, short‐term drug reactions, while clinical toxicity refers to the potentially harmful effects observed during real‐world clinical use. Both acute and clinical toxicity data assessments rely on in vivo data.

#### 2.1.1 Acute Toxicity

Acute toxicity of drugs is commonly evaluated through the determination of the lethal dose half (LD50) value. This assessment is typically conducted in mice or rats via oral, inhalation, or percutaneous exposure routes. The LD50 value serves as a foundational metric for the classification and labeling of drugs based on acute toxicity. Acute toxicity prediction can provide a reference basis for acute toxicity classification and labeling management of drugs, which is important for the preliminary estimation of target organ toxicity and underlying mechanisms. Commonly used acute toxicity datasets include TOXRIC, Integrated Chemical Environment (ICE), Environmental Protection Agency Distributed Structure‐Searchable Toxicity (EPA DSSTox)/EPA's ToxVal, ChemIDplus, eChemPortal, National Institute of Technology and Evaluation (NITE) Chemical Risk Information Platform, PubChem, and personalized datasets compiled by researchers.

Acute toxicity datasets often vary in quality and size, impacting the robustness of predictive models. Techniques such as data filtering and augmentation are employed to enhance dataset quality. Lou et al. integrated 1735 rabbit‐based and 1679 rat‐based experimental data. In the rat ML model, the RF model showed the best performance; ML often outperforms DL models due to insufficient amount of training data; RF performs best in traditional ML.^[^ ^11^ ^]^ They used SHAP to quantify the contribution of features to toxicity prediction, revealing key features affecting skin absorption (molecular weight, polarity, and lipophilicity). Using Attentive FP atomic heatmaps, they visually displayed the atomic‐level attention distribution, clearly showing the contributions of toxic‐related atoms and structure.

Bayesian inference and classification schemes like Cramer's Threshold of Toxicological Concern (TTC) aid in classifying chemicals based on their toxicity levels with higher certainty.^[^ ^12^ ^]^ Wijeyesakere et al. used ICE Rat Acute Toxicity data to train a RF Quantitative Structure‐Activity Relationships (QSAR) model, classifying chemicals based on LD50 into Globally Harmonized System of Classification and Labelling of Chemicals (GHS) categories. The model exhibited good sensitivity in predicting chemicals in GHS Category 1–2 or GHS Category 1–3.^[^ ^13^ ^]^

Effective representation of molecular structures is crucial for toxicity prediction research. MolFPG proposes a framework that combines molecular fingerprints with molecular graphs. By encoding chemicals using various molecular fingerprint techniques and integrating molecular representations based on Graph Transformer, the framework enables feature learning and toxicity prediction.^[^ ^11^, ^14^ ^]^ It incorporates local interpretability analysis by selecting molecular samples and calculating atomic attention weights, combining these with hidden layer states of a multi‐layer GNN.

Utilization of interpretable ML models and attention mechanisms helps in elucidating toxicity mechanisms. Ketkar et al. tested the application of multiple graph models in four virulence tasks (fish, Daphnia magna, Tetrahymena, and Vibrio fischeri).^[^ ^15^ ^]^ The models include Message Passing Neural Networks (MPNN), Graph Convolutional Networks (GCN), Generative Adversarial Network (GAN), Path‐Augmented Graph Transformer Network (PAGTN), and Attentive FP. Attentive FP was reported to have the lowest prediction error and the best performance in all four tasks. The attention weights of the Attention FP model provide good interpretability.

To summarize, acute toxicity is often assessed using endpoints like LD50 for regression prediction tasks, with few studies utilizing thresholds of LD50 for classification tasks. Bo et al found that better performance was achieved by utilizing data suitable for regression modeling.^[^ ^16^ ^]^ By using molecular graphs as input to drugs and employing GNNs to process these molecular graphs, the model's predictive performance is further enhanced, potentially capturing detailed toxicity features. The emergence of databases like ChemIDplus and TOXRIC has led to a rapid expansion of acute toxicity training data, reaching ≈100000 data points across multiple species. This allows for the training of more accurate DL regression models. However, data scarcity remains in human studies. Therefore, bridging the gap between animal models and human toxicity predictions is critical. Exploring new techniques such as Zero‐Shot Learning (ZSL) and Transfer Learning (TL) to predict acute toxicity in humans will be a key research focus.

#### 2.1.2 Clinical Toxicity

Clinical toxicity refers to the damage caused by a drug to human organs or tissues and is a clinical experiment conducted at the human level. Accurately and effectively predicting the safety of new drug candidates in humans is a great challenge. Predictions of in vitro, in vivo, or human clinical toxicity are not always consistent; in vitro, testing is the most fine‐grained and captures a chemical's ability to disrupt biological pathways at the cellular level. In contrast, clinical testing is coarse‐grained and captures chemical interactions at multiple levels in the human body, including organs and tissues, and ML models trained on in vitro and in vivo data may not reliably capture clinical toxicity. Commonly used datasets for clinical toxicity prediction are Tox21, ClinTox, and Registry of Toxic Effects of Chemical Substances (RTECS).

Expanding clinical toxicity studies can help optimize the pipeline for future toxicity screening experiments across various in vivo, in vitro, and clinical endpoints. Sharma et al. used a multi‐task DL framework to simultaneously model in vitro, in vivo, and clinical toxicity data.^[^ ^17^ ^]^ Morgan fingerprints and pre‐trained Simplified Molecular‐Input Line‐Entry System (SMILES) embeddings are used as input representation, which improves clinical toxicity prediction compared to existing models in the MoleculeNet benchmarks, with no improvement on the in vivo platform. Additionally, the results showed that clinical toxicity prediction requires minimal in vivo data, challenging the traditional stepwise validation sequence from in vitro to in vivo to human samples in molecular biology experiments. More studies are needed to explore the role of in vivo experiments in clinical toxicity prediction. Post hoc comparative interpretability analysis validated the rationality of the predictive features with known chemical reaction patterns. Post hoc comparative interpretability analysis validated the rationality of the predictive features with known chemical reaction patterns.

Due to limited clinical toxicity data, research in this area remains scarce. Clinical toxicity prediction still relies on in vitro and in vivo experimental data. The extrapolative relationships among these datasets require further investigation. Techniques like TL and ZSL offer the potential to improve predictive performance and uncover new insights.

### 2.2 Advances in Carcinogenicity Prediction

Carcinogenicity refers to the development of cancer caused by a drug or its metabolites, including anticancer agents, analgesics, and immunomodulators. For example, non‐oncology drugs with known carcinogenic potential include arsenic‐based preparation, natural estrogens, estradiol, finasteride, and non‐steroidal anti‐inflammatory drugs. Antineoplastic drugs, such as platinum‐based chemotherapeutic agents, hormone‐dependent tumor suppressors, and targeted agents, also carry a risk of carcinogenic effects. Despite stringent carcinogenicity testing requirements, several Food and Drug Administration (FDA) approved drugs have been determined to be carcinogenic and subsequently withdrawn from the market. In the United States, drug‐related cancer patients account for ≈20% of primary cancer cases.^[^ ^18^ ^]^ Therefore, it is essential to test new chemicals for carcinogenicity before they are released to the market. Datasets such as Drugbank, The Carcinogenic Potency Database (CPDB), Chemical Carcinogenesis Research Information System (CCRIS), and ISSCAN BIOVIA databases, are frequently utilized for the prediction of carcinogenicity.

Recent advancements in DL algorithms have enhanced predictive capabilities compared to traditional ML methods. Chen et al. introduced DCAMCP, a DL model leveraging capsule networks and attention mechanisms, specifically developed to discriminate between carcinogenic and non‐carcinogenic chemicals.^[^ ^19^ ^]^ Capsule networks capture hierarchical relationships within the data, while attention mechanisms highlight significant features, leading to enhanced model interpretability and accuracy. DCAMCP outperformed the traditional ML models such as K‐Nearest Neighbors (KNN), SVM, XGB, RF, and DNN.

Limited experimental data for model training and challenges in data collection and label representation hinder carcinogenicity prediction. Fradkin et al.^[^ ^20^ ^]^ investigated three orthogonal approaches to overcome dataset size limitations: architecture selection, dataset modification, and pre‐training techniques. They utilized a graph‐based representation, the GNN Transformer (GROVER), for molecular embeddings.^[^ ^21^ ^]^ Moreover, they evaluated the effectiveness of model pre‐training using two methods: TL with GROVER and multiple rounds of pre‐training on relevant but low‐quality data.

In molecular representation studies, latent embeddings generated by ML and natural language processing algorithms enhance carcinogenicity prediction performance. Mittal et al. constructed separate classification models for six distinct biochemical properties, such as electrophilicity and epigenetic modifications. They experimented with three representations—bioactivity‐based descriptors, chemistry‐based molecular descriptors, and graph‐based features—alongside various classification algorithms. After performance testing, Gradient Boosting Machines (GBM) are selected to predict carcinogens and non‐carcinogens. The Metabokiller ensemble model was created by combining six biochemical models with GBM‐based predictions and bootstrapping.^[^ ^22^ ^]^ Large‐scale computational screening of human metabolites in the Human Metabolome Data Bank (HMDB) database using Metabokiller identified many known and unknown human metabolites with carcinogenic potential. They also quantified the influence of biochemical processes, such as electrophilicity and oxidative stress, revealing potential biological mechanisms of carcinogenicity. To further enhance interpretability, the researchers applied the Local Interpretable Model‐Agnostic Explanations (LIME) algorithm to identify key biochemical features contributing to carcinogenicity. However, Metabokiller did not consider predicted dose information and biochemical properties of certain carcinogens, such as inhibition of senescence or impacts on signaling pathways, which is a direction for future consideration.

In summary, carcinogenicity prediction is typically framed as a classification task to distinguish carcinogenic from non‐carcinogenic chemicals. Future directions include representation learning using DL and strategies for heterogeneous data fusion. Additionally, the threshold dose of a drug and its impact on the immune microenvironment are critical considerations. Low‐dose carcinogens may have a latency threshold below which tumor progression can be suppressed.^[^ ^23^ ^]^ Microenvironmental markers, such as immunosuppressive cells and cytokines, can assist in carcinogenicity prediction.^[^ ^24^ ^]^ By integrating chemical properties, biological activity, and toxicological pathway data, multimodal models can combine molecular interactions and immune regulation to improve prediction accuracy and uncover underlying mechanisms. The accumulation of multi‐omics data derived from drug perturbations will further enhance the accuracy and interpretability of ML‐based carcinogenicity predictions.

### 2.3 Advances in Organ‐Specific Toxicity Prediction

Organ‐specific toxicity is crucial because drugs often target specific tissues or organs, leading to adverse effects due to local accumulation or organ‐specific susceptibility. Drug‐induced organ toxicity includes hepatotoxicity, cardiotoxicity, nephrotoxicity, respiratory toxicity, and neurotoxicity.

#### 2.3.1 Hepatotoxicity

The liver is the metabolic center of the body, and damage to the liver caused by the drug itself or/and its metabolite during administration is known as drug hepatotoxicity/drug‐induced liver injury (DILI). For more than 90% of orally administered drugs, the liver is the primary site of structure‐dependent metabolism. The conventional markers used to diagnose hepatotoxicity are aspartate aminotransferase (AST), alanine aminotransferase (ALT), alkaline phosphatase (ALP), serum concentrations of total bilirubin and albumin, and prothrombin time. However, the sensitivity and specificity of these biomarkers are not good and have limited prognostic value for possible liver damage. Another key issue during drug development is the species specificity of target organ toxicity, and even more difficult to identify DILI in clinical trials is the low concordance (≈50%) between DILI results in animal and human models.^[^ ^25^ ^]^ Considering the problems of biomarkers and species‐specific toxicity, the most valuable data on drug‐induced hepatotoxicity is obtained by combining case reports and literature data.^[^ ^26^ ^]^ There are many hepatotoxicity datasets, commonly used are ToxCast, Tox21, TOXRIC, Side Effect Resource (SIDER), Clintox, DILIrank, LiverTox, Hepatox, Liver Toxicity Knowledge Base (LTKB), ChEMBL, DrugBank, DILIRank, Therapeutic Target Database (TTD), Pharmacogenomics Knowledge Base (PharmGKB), Drug‐Induced Liver Injury Severity and Toxicity (DILIst), Therapeutic Data Commons (TDC), Toxicogenomics Project‐Genomics Assisted Toxicity Evaluation System (TG‐GATEs) and HMDB.

Chen et al. developed ResNet18DNN, a DNN model using composite structural images to improve hepatotoxicity prediction performance.^[^ ^27^ ^]^ Füzi et al. introduced a systems biology approach integrating descriptors related to biological pathways, showing superior performance in predicting hepatotoxicity compared to traditional ML models like Gradient Boosting Trees (GBT) and RF.^[^ ^28^ ^]^

Data imbalance and feature scarcity limit the performance of drug hepatotoxicity prediction. To address these issues, Çeli K et al proposed using binary classifiers in a one‐versus‐many (OvA) approach to simplify multiclass prediction, improving accuracy and efficiency.^[^ ^26^ ^]^ This study used heatmaps of descriptor importance and OvA classification methods to improve model interpretability. Lim et al. introduced supervised subgraph mining (SSM) to identify subgraph features associated with DILI, enhancing accuracy in structural alert (SA) identification and mechanism inference.^[^ ^29^ ^]^ These subgraph features provide the model with internal interpretability. Through SMARTS pattern matching and graphical abstraction, post‐processing interpretable analyses are performed to show the relationship between the subgraphs and DILI. However, balancing coverage and specificity in subgraph selection remains a challenge. In cases where activity data is unavailable, Füzi et al.’s pipeline combined with similarity‐based methods allows the creation of unique profiles for new chemicals by merging targets, interactomes, and pathway profiles.^[^ ^28^ ^]^

To explore the mechanisms of hepatotoxicity, Martínez‐Sena et al. utilized non‐targeted mass spectrometry and SVM models to identify metabolomic biomarkers in HepG2 cells, predicting hepatotoxicity mechanisms and enhancing predictive models for DILI.^[^ ^30^ ^]^ Using metabolomics strategies, quantitative toxicity indicators, and metabolic pathway analysis, the model can predict whether a chemical is hepatotoxic and provide related toxicity mechanisms, such as oxidative stress, mitochondrial damage, cell apoptosis, and steatosis. Lee et al. used RF, Light Gradient Boosting Machine (LGBM), LR, and Neural Network (NN) models to interpret DILI prediction results, identifying key molecular descriptors and using feature importance rankings and LR coefficients to improve interpretability.^[^ ^31^ ^]^ Chen et al developed Tox‐GAN, a GAN‐based framework generating transcriptomic profiles to simulate drug toxicity outcomes,^[^ ^32^ ^]^ providing local interpretability through gene‐level, functional‐level, biomarker‐based, and pathway‐based analysis on transcriptomic data. Jin et al. applied the entropy weight method (EWM) to assess the degree of pathway perturbation based on the gene expression profiles under DILI conditions: the higher the dispersion of gene expression, the higher the contribution of the gene to pathway perturbation, and the higher the gene contribution.^[^ ^33^ ^]^

In summary, drug hepatotoxicity prediction is often framed as a classification task, with data quality issues complicating the determination of DILI‐free drugs. Factors like dosage, frequency, drug prevalence, and patient susceptibility can influence outcomes. Additionally, some drugs may cause liver damage indirectly, such as by inhibiting bile salt export. Incorporating in vitro data (mitochondrial toxicity, bile salt export pump inhibition) and in vivo data (preclinical rat liver toxicity studies) can improve the performance of models.^[^ ^34^ ^]^ Future predictions should consider the immune microenvironment, species differences, and gut microbiome influence for more accurate hepatotoxicity prediction in drug development.

#### 2.3.2 Cardiotoxicity

Drug cardiotoxicity refers to the toxic effects of a drug on the myocardium, including electrophysiologic cardiotoxicity (ECT) and structural cardiotoxicity (SCT). ECT is characterized by drug‐induced abnormalities in cardiac function and ion channel activity, often leading to prolonged QT intervals on electrocardiograms and fatal ventricular arrhythmias. Studies on ECT commonly focus on the response of voltage‐gated potassium ion channels, particularly human ether‐related genes (hERG). SCT is clinically defined as a change in left ventricular ejection fraction, which may lead to fibrosis, cardiomyopathy, heart failure, and death. The pharmaceutical industry has suffered significant losses due to cardiotoxicity in the early, preclinical, or clinical stages of drug development, resulting in several drugs being withdrawn from the market and many drug development programs being halted in their pipelines. AI‐assisted drug toxicity discovery is an effective solution to reduce costs and facilitate the development of lead drug candidates. The most used cardiotoxicity datasets are Tox21, ChEMBL, ICE, PubChem, BindingDB, and hERGCentral.

For ML methods, the RF model achieves good performance. Krishna et al. integrated hERG bioactivity data from the ChEMBL database with data from the Tox 21 qHTS thallium flux assay for the prediction of cardiotoxicity. The RF approach yielded 92.6% equilibrium accuracy, and it allowed for the analysis of which descriptors had the greatest impact on model performance.^[^ ^35^ ^]^ Yang et al. linked atomic‐scale information to protein, cellular, and tissue scales by predicting drug binding affinities and rates from ion channel simulations.^[^ ^36^ ^]^ These values were used in cell‐ and tissue‐scale models to predict drug effects on hERG channels. The framework was validated with human clinical data, demonstrating good agreement and revealing complex interactions between arrhythmia mechanisms and potential emergent behaviors. The RF model was used to analyze the importance of parameters related to arrhythmia susceptibility for interpretability.

For DL methods, Luca et al. explored chemical interactions within the AOP network from Molecular Initiation Events (MIE) to Key Events (KE).^[^ ^37^ ^]^ They used various encoding methods, from traditional QSAR techniques to graph‐based methods, autoencoders, and Natural Language Processing (NLP)‐like character embeddings. Their multimodal NN showed improved performance with increased training data. The NLP model with data augmentation outperformed the baseline XGB model, demonstrating the potential of DL models to enhance prediction accuracy by learning from varied chemical representations. This study also involved analysis using SHAP and permutation importance methods for model interpretation.

Wang et al. developed the DMFGAM model, which fuses multiple molecular fingerprints with a multi‐head GAT model to extract molecular graph features.^[^ ^38^ ^]^ These were used to classify molecules as hERG blockers or non‐blockers via a fully connected NN. Arab et al. developed a DL framework to predict cardiotoxicity affecting three cardiac ion channels: hERG, Cav1.2, and Nav1.5.^[^ ^39^ ^]^ Their study rigorously compared three types of property representations—molecular fingerprints, descriptors, and graph‐based numerical representations. Au Yeung et al. identified 52 potential biomarkers associated with SCT by using a human in vitro cardiac organoid model combined with transcriptomics and ML approaches.^[^ ^40^ ^]^ This study integrated transcriptomics and biological networks to elucidate the molecular mechanisms predicted by the model, providing a global interpretability analysis.

In summary, the cardiotoxicity of drugs in humans can be reflected by electrophysiologic changes in cardiac enzymes, electrocardiogram, and cardiac ultrasound. The prediction task is often a classification task, determining whether a drug has cardiotoxicity. Utilizing traditional blood biomarkers such as troponin and B‐type natriuretic peptides (BNPs), which have high specificity, in combination with novel markers of cardiomyocyte injury due to drug perturbation and multi‐omics data, the selection of a suitable AI model based on the amount of data can facilitate further improvement of the accuracy and interpretability of cardiotoxicity prediction. At the same time, the role of non‐cardiomyocytes (70% of the total heart) in cardiotoxicity cannot be ignored.

#### 2.3.3 Nephrotoxicity

Nephrotoxicity is the deterioration of renal function due to the toxic effects of a drug. The kidneys play a crucial role in human physiology, responsible for excreting waste products, regulating fluid balance, maintaining electrolyte levels, and stabilizing hormone concentrations. They are particularly susceptible to drug toxicity compared to other organs. Therefore, it is important to develop a mechanism to optimally and rapidly analyze the nephrotoxicity of drug candidates. Commonly used nephrotoxicity datasets are SIDER, Open TG‐GATEs, and Toxygates.

DL and ML might demonstrate varied predictive performances across datasets. Mazumdar et al. explored the predictive abilities of DL and ML models on a dataset comprising 287 nephrotoxic and 278 non‐nephrotoxic drugs.^[^ ^41^ ^]^ Their findings indicated that DNN excelled in generalization during quintuple cross‐validation, while the Extra‐tree model outperformed on test data. Molecular biology‐based models also surpassed fingerprint mapping‐based models. The study used association rule mining to identify key substructures associated with nephrotoxic chemicals and employed support and confidence analyses to explain the relationship between structures and toxicity.

Genomics data have emerged as effective molecular representations in predicting nephrotoxicity. Su et al. developed Att‐RethinkNet, a novel multi‐label learning model designed to predict drug‐induced liver and kidney pathological outcomes using toxicogenomic data.^[^ ^42^ ^]^ Instead of conventional toxicity classification, Att‐RethinkNet employs a memory structure to effectively utilize label association information. It integrates attentional mechanisms to enhance the presentation of crucial features, making it versatile for predicting hepatotoxicity and nephrotoxicity across different organs. However, the model's dependence on manually labeled pathology findings restricts its applicability to datasets with large label collections or a high number of drugs.

In summary, nephrotoxicity is typically assessed through pathological results, which are used to create binary classification training data. However, due to the difficulty in obtaining pathological data, there is a limited amount of labeled data for nephrotoxicity. As a result, most studies rely on the chemical structure and genomic features of drugs for characterization. Blood and urine kidney function markers are relatively easy to obtain and provide valuable labels for nephrotoxicity prediction. Using clinical data and electronic health records, ML models were able to provide an accurate prediction of acute kidney injury by learning and analyzing patterns of data.^[^ ^43^ ^]^ As new DL models continue to be developed, integration of multi‐modal data (e.g., images and physiological parameters) will be able to improve nephrotoxicity prediction performance.

#### 2.3.4 Respiratory Toxicity

Respiratory toxicity is defined as respiratory or lung damage resulting from inhalation of a drug from the respiratory tract or through other routes to the lungs. Generally, the adverse effects of common drugs on the human respiratory system are not apparent in the initial stages, and it is critical to establish methods for evaluating the potential respiratory toxicity of chemicals as early as possible in the drug development process. Commonly used datasets for respiratory toxicity prediction are ChemIDplus, PNEUMOTOX, ADReCS, Hazardous Chemical Information System, SIDER, and IntSide databases.

Zhang et al. used a genetic algorithm to screen important molecular descriptors related to respiratory toxicity and applied the ECFP6 fingerprint as input.^[^ ^44^ ^]^ The overall prediction accuracy of the training and external test sets generated by the Naive Bayes (NB) classifier was 91.8% and 84.3%, respectively. They explained model predictions through descriptors and fingerprint fragment analysis and discussed limitations by examining misclassified chemicals. Lei et al. used a four‐layer dimensionality reduction strategy to find 20 optimal molecular descriptors for mouse intraperitoneal respiratory toxicity.^[^ ^45^ ^]^ The results showed that SVM preforms best in the regression task and XGB preforms best in the classification task. Jaganathan et al. utilized eight ML algorithms for respiratory toxicity prediction, with SVM emerging as the top performer.^[^ ^9^ ^]^ Using the SHAP method, they provided global and local explanations, revealing the importance of E‐state and topological descriptors in enhancing predictive accuracy.

In summary, fewer DL models are applied to respiratory toxicity prediction, which may be related to the small amount of respiratory toxicity data based on drug perturbations. Fully exploiting the available data may facilitate the prediction performance of the models. For example, using marketed drugs as negative samples can improve the prediction performance of the binary classification “co‐toxicity” task, and also enhance the stability of model prediction.^[^ ^46^ ^]^ With the accumulation of respiratory toxicity data, the utilization of DL models is expected to further improve the performance of respiratory toxicity prediction. Additionally, to develop more reliable predictive models for respiratory toxicity, experimental data related to more respiratory symptoms or endpoints (such as bronchitis, rhinitis, or pneumonia) should be used than for respiratory sensitization.

#### 2.3.5 Neurotoxicity

Neurotoxicity is any adverse effect of a drug on the structural or functional integrity of the central nervous system, peripheral nerves, or sense organs. Many clinical drugs can cause neurotoxicity, which has been a global human concern. Neurotoxic and non‐neurotoxic drugs often exhibit distinct physicochemical properties. There are fewer neurotoxicity datasets, and commonly used datasets for neurotoxicity prediction are SIDER and PubChem.

Advancements in high‐content imaging have enabled improved phenotyping of brain‐like organoids. Monzel et al. developed an RF model for predicting toxicity in brain organoids treated with 6‐hydroxydopamine, utilizing image‐based cellular analysis.^[^ ^47^ ^]^ They demonstrated RF's effectiveness in predicting neurotoxicity perturbations, incorporating variable importance assessments and principal component analysis for enhanced interpretability. Another study proposed that 2D models are more robust under stringent genomic selection conditions, and the accuracy of 3D models decreases dramatically.^[^ ^48^ ^]^ However, 3D models of organoids are closer to the real tissue microenvironment and are expected to further facilitate the prediction of drug‐induced neurotoxicity as technology and algorithms advance.

Identifying structural features of chemical neurotoxicity facilitates the early design of non‐toxic chemicals. Zhao et al. extracted drug neurotoxicity data from humans during real‐world clinical use and developed 35 different classifiers by combining five different ML methods and 7 fingerprinting, with the MACCS‐SVM model performing best.^[^ ^49^ ^]^ They identified 18 structural alarms (SA) associated with neurotoxicity and provided interpretable insights.

In conclusion, the main obstacle to developing DL prediction models is the limited data on neurotoxicity, and studies have been conducted to find new methods to process the existing data while continuously accumulating new data. Neurotoxic peptides have structural diversity, targeting, and great medicinal potential. Lee et al proposed a peptide data augmentation method based on random substitution or insertion of arbitrary amino acids in known neurotoxic peptides to expand the data, which improves neurotoxic peptide identification by the Convolutional Neural Network (CNN) model.^[^ ^50^ ^]^

### 2.4 Advances in Cellular and Molecular Toxicity Prediction

Cellular and molecular toxicity refers to the toxicity caused by drugs directly affecting the structure and function of cells or subcellular organelles. This section primarily focuses on hematotoxicity and mitochondrial toxicity.

#### 2.4.1 Hematotoxicity

Hematotoxicity is the direct cytotoxicity of a drug to circulating mature blood cells or immature hematopoietic stem/progenitor cells in the bone marrow. Drug‐induced hematologic disorders occur less frequently than other adverse reactions but are associated with a high mortality rate. Chemical‐induced hematotoxicity is currently assessed mainly by in vivo experiments such as bone marrow aspiration in animal species. Considering the ethical issues, and high economic and time costs of experimental analyses, the development of reliable computational models for predicting hemotoxic chemicals is essential. There are fewer studies of AI modeling predictions of hematotoxicity and a lack of specific hematotoxicity databases, such as Tox21 or the National Center for Advancing Translational Sciences (NCATS). Insufficient data and interpretive uncertainty add to the difficulty of developing reliable predictive models of hematotoxicity. Commonly used datasets for hematotoxicity prediction are SIDER, The Online Chemical Modeling Environment (OCHEM), and PubChem Bioassay.

To overcome the challenge of data scarcity, Long et al. constructed a high‐quality dataset containing 759 hematotoxic and 1623 non‐hematotoxic chemicals.^[^ ^51^ ^]^ They built a series of classification models based on a combination of seven ML algorithms and nine molecular representations. Results based on two data partitioning strategies and domain‐of‐applicability analyses showed that Attentive FP‐based predictive models performed best. The study used SHAP to evaluate the marginal contribution of features and atomic heatmaps.

Leveraging real patient data allows researchers to directly explore and identify risk factors for hematologic toxicity. To identify risk factors and predict the likelihood of Bruton's tyrosine kinase inhibitor (BTKi)‐related hematologic toxicity, Jiang et al. constructed and validated a predictive model for hematologic toxicity of BTKi.^[^ ^52^ ^]^ They collected real data from patients treated with BTKi at the China Lymphoma Research Center, and 36 candidate variables were categorized as demographics, diagnostic and treatment information, laboratory data, and medical history. They developed and compared the performance of the ML methods and found that the XGB model demonstrated high discrimination and a profound association between these variables and hematologic toxicity. SHAP analysis is used to reveal the relationship between variables and hematologic toxicity.

Obtaining more high‐quality hematotoxicity data through data integration and sharing platforms, introducing multi‐omics data for comprehensive analysis, and using advanced DL technologies can help construct more complex and accurate haematotoxicity prediction models.

#### 2.4.2 Mitochondrial Toxicity

Mitochondria are known as the power stations of the cell, and mitochondrial toxicity refers to chemicals that cause signaling such as ER stress, oxidative stress, proteotoxic stress, or apoptosis within the cell, and may lead to cell death. The risk of mitochondrial toxicity in drug discovery can be indicated by experimental methods (e.g., Glu/Gal assays10) or by prediction methods trained using in vitro assay data. Commonly used datasets for mitochondrial toxicity prediction are Tox21, Mitotox, Gene Ontology, PubChem, and DrugBank.

Seal et al. aimed to predict mitochondrial toxicity by integrating cell painting morphology, gene expression data, and chemical structure information from Morgan fingerprints.^[^ ^53^ ^]^ Their approach improved predictive performance compared to models using only chemical structure, highlighting the importance of integrating biological data for accurate toxicity prediction. The study used local interpretability analysis, employing feature selection, correlation analysis, and single DT classifiers to reveal key features and their biological significance in the specific prediction task. Jaganathan et al. developed an interpretable tree‐based ML model to classify chemicals as mitochondrial toxic or non‐toxic, focusing on molecular descriptors.^[^ ^54^ ^]^ They found that Mordred feature descriptors and the CatBoost model outperformed other methods in classification accuracy. SHAP value analysis and tree‐based ensemble models enhanced the global interpretability of the model.

Due to the small dataset of mitochondrial toxicity, there are fewer studies applying DL methods to predict mitochondrial toxicity. With the accumulation of data, the application of DL and more molecular representations will further facilitate the accurate prediction of mitochondrial toxicity. Toxicity prediction is moving toward greater accuracy, and toxicity prediction at the subcellular level facilitates further explanation of the mechanisms of drug toxicity.

**Figure 3:**
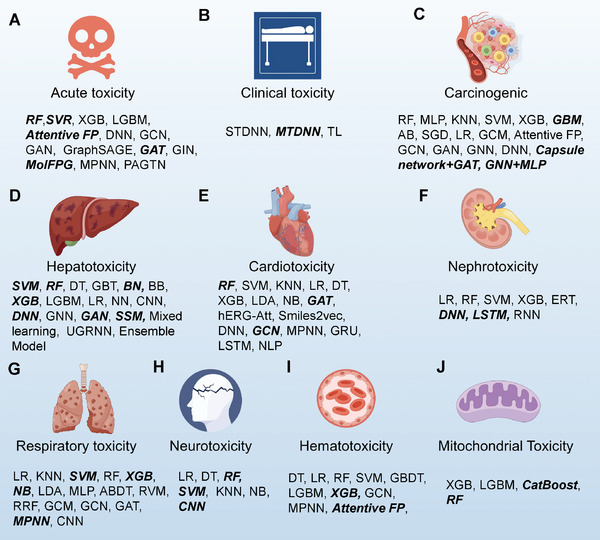
Applications of ML in Drug Toxicity Prediction. The figure shows various ML models used for predicting different types of drug toxicity, highlighting specific algorithms used for each toxicity category. It provides a strategic reference for selecting different ML models based on the nature of the toxicity type and the available data. A) Acute Toxicity: RF, SVR, Attentive FP, GAT, and MolFPG performed well in specific acute toxicity prediction studies. B) Clinical Toxicity: MTDNN showed good performance in predicting specific clinical toxicity. C) Carcinogenicity: GBM, Capsule network + GAT, and GNN + MLP performed well in specific carcinogenicity prediction studies. D) Hepatotoxicity: SVM, RF, BN, XGB, DNN, GAN, and SSM performed well in specific hepatotoxicity prediction studies. E) Cardiotoxicity: RF, GAT, and GCN showed good performance in specific cardiotoxicity prediction studies. F) Nephrotoxicity: DNN and LSTM were effective in predicting specific nephrotoxicity. G) Respiratory Toxicity: SVM, XGB, NB, and MPNN performed well in specific respiratory toxicity prediction studies. H) Neurotoxicity: RF, SVM, and CNN showed good performance in specific neurotoxicity prediction studies. I) Hematotoxicity: XGB and Attentive FP performed well in specific hematotoxicity prediction studies. J) Mitochondrial Toxicity: Catboost and RF showed good performance in specific mitochondrial toxicity prediction studies. The bolded italicized portions are algorithms that have achieved good performance in a particular study.

Finally, we summarized representative ML and DL algorithms (Figure 2), along with the performance results of all published algorithms (**Table** **1**) and the optimal ones in each toxicity category (**Figure** **3** and Table S1, Supporting Information), to facilitate researchers in conducting studies in the corresponding toxicity areas. Among the algorithms, RF, SVM, and XGB are traditional ML models. RF has performed well in drug toxicity prediction tasks, especially in large‐scale datasets, achieving notable results in predicting acute toxicity, hepatotoxicity, cardiotoxicity, neurotoxicity, and mitochondrial toxicity (Figures 2, 3). SVM is widely used to predict various toxicity‐related endpoints, and it has shown strong performance in hepatotoxicity, respiratory toxicity, and neurotoxicity prediction (Figures 2, 3). XGB, which combines multiple weak learners (such as DTs) to form a strong model, is particularly suited for handling complex nonlinear relationships. It has shown good performance in predicting hepatotoxicity, respiratory toxicity, and hematotoxicity (Figures 2C, 3D, G, I).

**Table 1:** Performance scores and validation scheme of some methods involved in this review.

Number	Class	Category	Study	Algorithms	Validation scheme	Classification performance								Regression performance		
						AUROC	ACC	F1	MCC	Precision	Sensitivity	Specificity	Recall	MSE	RMSE	R^2^
1	Acute and Clinical Toxicity	Acute Toxicity	Lou et al.^[^ ^11^ ^]^	RF, SVM, XGB, LGB, GCN, GAN, Attentive FP, **GAT**	10‐fold cross‐validation	0.82	0.763		0.73							
			Firman et al.^[^ ^12^ ^]^	**RF**	10‐fold cross‐validation											0.739
			Wijeyesakere et al.^[^ ^13^ ^]^	**RF**							0.766	0.882				
			Teng et al.^[^ ^14^ ^]^	RF, XGB, Attentive FP, GCN, GraphSAGE, GAT, GIN, **MolFPG**		0.868									0.679	
			Ketkar et al.^[^ ^15^ ^]^	MPNN, GCN, GAT, PAGTN, **Attentive FP**	5‐fold cross‐validation									0.183		0.835
			Bo et al.^[^ ^16^ ^]^	**SVR**, RF, XGB, DNN	5‐fold cross‐validation	0.77	0.65								0.28	0.77
		Clinical toxicity	Sharma et al.^[^ ^17^ ^]^	STDNN, **MTDNN**, TL	5‐fold cross‐validation	0.994	0.963									
2	Carcinogenicity	Carcinogenicity	Chen et al.^[^ ^19^ ^]^	KNN, SVM, XGB, RF, DNN, **capsule network+GAT**	5‐fold cross‐validation	0.793	0.718									
			Fradkin et al.^[^ ^20^ ^]^	RF, GNN, AB, **GNN+MLP**	3‐fold cross‐validation	0.73								0.71		
			Mittal et al.^[^ ^22^ ^]^	LR, RF, MLP, KNN, SVM, SGD, GCM, attentive FP, GCN, GAN, **GBM**	10‐fold cross‐validation	0.98	0.94									
3	Organ‐specific Toxicity	Hepatotoxicity	Çeli K et al.^[^ ^26^ ^]^	**BN**, DT, RF	10‐fold cross‐validation		0.893			0.891						
			Lim et al.^[^ ^27^ ^]^	GNN, **SSM**		0.691	0.687	0.784	0.338							
			Füzi et al.^[^ ^28^ ^]^	DT, **RF**, GBDT	5‐fold cross validation		0.766			0.769	0.73	0.799				
			Chen et al.^[^ ^29^ ^]^	CNN, **DNN**, BB, SVM, RF, Mixed Learning, UGRNN, Ensemble Model		0.978	0.978		0.957		1	0.957				
			Martínez‐Sena et al.^[^ ^30^ ^]^	**SVM**	3‐fold cross‐validation	0.8										
			Lee et al.^[^ ^31^ ^]^	LR, **RF**, LGBM, NN	10‐fold cross‐validation	0.97	0.9	0.89		0.83	0.96	0.87				
			Chen et al.^[^ ^32^ ^]^	DNN, **GAN**		0.973	0.992	0.995		0.994		0.975	0.995			
			Jin et al.^[^ ^33^ ^]^	**XGB**			0.63			0.91		0.89	0.51			
																
		Cardiotoxicity	Krishna et al.^[^ ^35^ ^]^	DNN, **RF**, SVM, LDA	10‐fold cross‐validation		0.999		0.997		0.999	0.996				
			Yang et al.^[^ ^36^ ^]^	**RF**			0.98									
			Luca Viganò et al.^[^ ^37^ ^]^	LR, DT, RF, SVM, KNN, XGB, NB, MPNN, GRU, LSTM, **NLP**	k‐fold cross‐validation		0.815	0.687	0.585	0.616	0.777	0.883				
			Wang et al.^[^ ^38^ ^]^	RF, **GAT**, Smiles2vec, hERG‐Att, GCN	5‐fold cross‐validation	0.894	0.817		0.63							
			Arab et al.^[^ ^39^ ^]^	**GCN**			0.864	0.901	0.702		0.981	0.778				
			Yeung et al.^[^ ^40^ ^]^	**RF**		0.8				0.88			0.68			
		Nephrotoxicity	Mazumdar et al.^[^ ^41^ ^]^	XGB, ERT, **DNN**	5‐fold cross‐validation	0.878	0.821	0.86	0.644		0.804	0.865				
			Su et al.^[^ ^42^ ^]^	LR, RF, SVM, RNN, **LSTM**	5‐fold cross‐validation	0.99	0.975	0.992			0.991	0.995				
		Respiratory toxicity	Zhang et al.^[^ ^44^ ^]^	**NB**	5‐fold cross‐validation	0.884	0.918				0.902	0.944				
			Lei et al.^[^ ^45^ ^]^	RVM, **SVM**, RRF, **XGB**, NB, LDA	leave‐one‐out cross validation	0.893	0.826		0.644		0.832	0.822			0.4	0.743
			Jaganathan et al.^[^ ^9^ ^]^	LR, KNN, **SVM**, RF, XGB, NB, MLP, ABDT	10‐fold cross‐validation		0.862		0.722		0.879	0.849				
			Zhou et al.^[^ ^46^ ^]^	RF, SVM, GCM, GCN, GAT, MPNN, CNN		0.966										
	
		Neurotoxicity	Monzel et al.^[^ ^47^ ^]^	**RF**	5‐fold cross‐validation		0.93									
			Kuusisto et al.^[^ ^48^ ^]^	SVM, LR, **RF**, NB		>0.9										
			Zhao et al.^[^ ^49^ ^]^	RF, **SVM**, KNN, NB, DT	5‐fold cross‐validation	0.835	0.765		0.53		0.765	0.765				
			Lee et al.^[^ ^50^ ^]^	**CNN**	5‐fold cross‐validation		0.995	0.995		0.992			0.998			
4	Cellular and Molecular Toxicity	Hematotoxicity	Long et al.^[^ ^51^ ^]^	RF, XGB, SVM, GBDT, GCN, MPNN, **Attentive FP**		0.768	0.726	0.663	0.449							
			Jiang et al.^[^ ^52^ ^]^	LR, DT, RF, GBDT, **XGB**, LGB,		0.671	0.73				0.467	0.913				
														
		Mitochondrial Toxicity	Seal et al.^[^ ^53^ ^]^	**RF**	4‐fold nested cross‐validation		0.78	0.47			0.79					
			Jaganathan et al.^[^ ^54^ ^]^	XGB, LGB, **CatBoost**, RF	10‐fold cross‐validation	0.92	0.871	0.855			0.831	0.905				

Note: The bolded portions are algorithms that have achieved good performance in a particular study. The abbreviations appearing for the first time in the table are as follows: AUROC (Area Under the Receiver Operating Characteristic Curve), ACC (Accuracy), F1 (F1 Score), MCC (Matthews Correlation Coefficient), MSE (Mean Squared Error), RMSE (Root Mean Squared Error); SVR (Support Vector Regression), GraphSAGE (Graph Sample and Aggregate), GIN (Graph Isomorphism Network), PAGAN (Position‐Aware Graph Attention Network), STDNN (Single‐Task Deep Neural Networks), AB (Adaptive Boosting), GCM (Graph Convolutional Model), BN (Bayesian Network), BB (Binary Bayesian), UGRNN (Unidirectional Gated Recurrent Neural Network)), LDA (Linear Discriminant Analysis), GRU (Gated Recurrent Unit), ERT (Extremely Randomized Tree), RVM (Relevance Vector Machine), RRF (Regularized Random Forest).

DNN, MTDNN, GNN, and GAT are typical DL algorithms. DNN requires high‐quality input data and is well‐suited for handling large‐scale, high‐dimensional data. It has demonstrated good performance in hepatotoxicity and nephrotoxicity prediction tasks (Figures 2, 3). MTDNN is an extended DNN that supports multi‐task learning, enabling the simultaneous prediction of multiple toxicity endpoints. By sharing underlying features and task‐specific layers, it enhances the model's overall learning ability for related tasks. MTDNN is applicable for joint prediction of multiple toxicity endpoints, such as clinical toxicity (Figures 2, 3). GNN is a DL model specifically designed to process graph‐structured data (e.g., molecular graphs), learning the relationships between nodes (atoms) and edges (bonds) in the molecular structure. It captures molecular features through a message‐passing mechanism and has been used in carcinogenicity and hepatotoxicity prediction (Figures 2, 3). GAT, a variant of GNN, integrates an attention mechanism that assigns different weights to nodes and edges in graph‐structured data, enhancing the model's focus on key molecular fragments. GAT is suitable for learning complex interactions between molecules, especially in predicting acute toxicity, and cardiotoxicity (Figures 2, 3). The GNN andMLP combination utilizes molecular graph embeddings extracted by GNN as inputs, which are then passed to an MLP for further predictions. This approach combines the molecular feature extraction capability of GNN with the strong classification/regression power of MLP, achieving good performance in carcinogenicity prediction (Figures 2, 3). LSTM, an improved version of RNN, focuses on handling sequential data and can capture long‐term dependencies. LSTM has shown strong performance in nephrotoxicity prediction (Figures 2, 3).

## 3 Databases and Tools for ML‐Enabled Drug Toxicity Prediction

**Figure 4:**
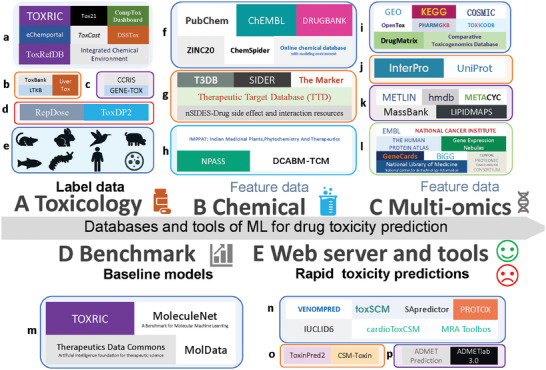
Databases and tools of ML for drug toxicity prediction A) Toxicity Databases provide label data for drug toxicity prediction: a. Universal Chemical Toxicity Database, b. Organ‐Specific Databases, c. Carcinogenicity Database, d. Other Toxicity Databases, e. Toxicity Databases covering rodents, rabbits, birds, pigs, fish, insects, humans, and in vitro cell levels. B) Chemical Databases provide feature data for drug toxicity prediction: f. Universal Chemical and Drug Databases, g. Chemical‐related Target and Interaction Databases, h. Natural Product and Traditional Medicine Databases. C) Multi‐Omics Databases provide feature data for drug toxicity prediction: i. Genomic and Transcriptome Databases, j. Proteomics Databases, k. Metabolomics Databases, l. Multi‐Omics Databases. D) ML Benchmark Databases provide ML benchmark results for drug toxicity prediction: m. ML Benchmark Databases. E) Web Servers and Tools provide rapid visualization, analysis, and quantification of chemical toxicity predictions: n. Chemical Toxicity Prediction Tools, o. Protein and Peptide Toxicity Prediction Tools, p. ADMET Prediction Tools.

Accurate and reliable data must be the foundation for building reliable AI‐driven models. Here, we list the databases and tools used in toxicity prediction‐related studies and classify the databases into 4 categories, where toxicology databases are used to provide toxicity data, which are often used as labeled data for toxicity prediction. Chemical and omics databases are utilized to acquire information and features of chemicals, which are used as input for predictive models. The benchmark databases guide researchers in selecting appropriate feature types and baseline models for different endpoint datasets.^[^ ^55^ ^]^ User‐friendly web servers and tools directly provide biologists and clinicians with simple and fast prediction results. Comprehensive labels, feature databases, and tools will enable researchers in a variety of fields to understand toxicity prediction studies and determine the data to be used.^[^ ^55^ ^]^ We characterize the role of these databases in toxicity prediction, including 15 toxicity databases, 14 chemical databases, 23 omics databases, 4 benchmarking databases, and 12 web servers and tools (**Figure** **4** and **Table** **2**).

**Table 2:** Databases and tools used for chemical toxicity prediction.

Number	Database Categories	Database contents	Databases	Description	Safety Information		Molecular structure	Molecular activity		Omics				Single‐cell RNA‐seq	ML benchmarks	Website	Last update
					Toxicities	Side effect		Target	Pathway	Genome	Transcriptome	Proteome	Metabolome				
1	**Toxicology**	**Universal Chemical Toxicity Databases**	TOXRIC	Toxicological data and benchmarks	√		√	√			√				√	https://toxric.bioinforai.tech/	2022
2			Tox21	Chemicals, pesticides, food additives/contaminants	√											https://tox21.gov/	2013
3			Toxcast	Ordered chemical information	√											https://www.epa.gov/chemical‐research/exploring‐toxcast‐data	2023
4			CompTox Dashboard	Chemistry, toxicity, and exposure information	√											https://comptox.epa.gov/dashboard/	2023
5			DSSTox	Distributed Structure‐Searchable Toxicity Database	√		√									https://www.epa.gov/chemical‐research/distributed‐structure‐searchable‐toxicity‐dsstox‐database	2024
6			eChemPortal	Chemical property, and by GHS classification	√		√									https://www.echemportal.org/echemportal/	2023
7			ToxRefDB	In vivo	√			√								https://github.com/USEPA/CompTox‐ToxRefDB	2023
8			ICE	In vivo, in vitro toxicity, predicted toxicity	√		√									https://ice.ntp.niehs.nih.gov/	2024
9		**Organ‐Specific Databases**	ToxBank	Chemical Toxicity	√		√				√					https://toxbank.net/	2013
10			LTKB	Liver Toxicity	√										√	https://www.fda.gov/science‐research/bioinformatics‐tools/liver‐toxicity‐knowledge‐base‐ltkb	2022
11			LiverTox	Clinical and Research Information on DILI	√											https://www.ncbi.nlm.nih.gov/books/NBK547852/	2022
12		**Carcinogenicity Databases**	CCRIS	Carcinogenesis	√		√									https://pubchem.ncbi.nlm.nih.gov/source/22070	2018
13			GENE‐TOX	Genetic toxicology (mutagenicity) test data	√											https://pubchem.ncbi.nlm.nih.gov/source/22071	2018
14		**Other Toxicity Databases**	RepDose	Subacute to chronic toxicity	√		√									https://cefic‐lri.org/toolbox/repdose/	2023
15			ToxDP2	Dietary polyphenols	√											http://14.139.62.46/toxdpp/	2021
																	
1	**Chemical**	**Universal Chemical and Drug Databases**	PubChem	Chemical information	√	√	√	√	√							https://pubchem.ncbi.nlm.nih.gov/	2023
2			DrugBank	Drug knowledge	√	√	√	√	√							https://go.drugbank.com/	2023
3			ChEMBL	Drug discovery platform			√	√			√					https://www.ebi.ac.uk/chembl/	2024
4			ZINC20	Ultralarge‐Scale Chemical Database			√	√								https://zinc20.docking.org/	2023
5			ChemSpider	Chemical structure database			√									https://www.chemspider.com/	2020
6			OCHEM	Chemical and biological data	√		√									https://ochem.eu/home/show.do	2023
7		**Chemical related Target and Interaction Databases**	T3DB	Pollutants, pesticides, drugs and food toxins	√			√								http://www.t3db.ca/	2020
8			Therapeutic Target Database (TTD)	Therapeutic Target			√	√	√							https://db.idrblab.net/ttd/	2023
9			nSides	Side effects of drugs identified but not listed on the official FDA labeling		√										https://nsides.io/	2022
10			SIDER	Side Effect Resource		√			√							http://sideeffects.embl.de/	2015
11			TheMarker	Therapeutic biomarker, safety biomarkers	√			√								http://themarker.idrblab.cn/	2023
12		**Natural Product and Traditional Medicine Databases**	IMPPAT (2.0)	Phytochemicals of Indian medicinal plants	√		√									https://cb.imsc.res.in/imppat/home	2022
13			NPASS	Natural Product			√	√								https://bidd.group/NPASS/	2023
14			DCABM‐TCM	Constituents Absorbed into Blood and Metabolites of Traditional Chinese Medicine			√						√			http://bionet.ncpsb.org.cn/dcabm‐tcm/#/Home	2023
																	
1		**Genomic and transcriptome data**	Open TG‐GATEs	Toxicogenomics	√						√					https://www.opentox.net/open‐tg‐gates‐data‐access‐opentox	2015
2			PharmGKB	PHARMACOGENOMICS		√	√		√		√					https://www.pharmgkb.org/	2023
3			ToxicoDB	Toxicogenomics	√		√	√	√							https://www.toxicodb.ca/	2022
4			DrugMatrix	Toxicogenomics	√						√					https://norecopa.no/3r‐guide/drugmatrix	2022
5	**Omics**		CTD	Toxicogenomics	√		√	√	√							https://ctdbase.org/	2023
6			GEO							√						https://www.ncbi.nlm.nih.gov/geo/	2024
7			KEGG					√	√	√						https://www.genome.jp/kegg/	2024
8			COSMIC							√						https://cancer.sanger.ac.uk/cosmic	2024
9		**Proteomics**	InterPro									√				https://www.ebi.ac.uk/interpro/	2024
10			UniProt				√					√				https://www.uniprot.org/	2024
11		**Metabolomics**	METLIN				√						√			https://massconsortium.com/index‐xcms‐metlin.html	2023
12			HMDB										√			https://hmdb.ca/	2022
13			MssBank										√			https://massbank.eu/MassBank	2023
14			LIPID MAPS				√						√			https://lipidmaps.org/	2022
15			MetaCyc/BioCyc						√	√			√			https://metacyc.org/	2023
16		**Multi‐omics**	EMBL_EBI							√				√		https://www.ebi.ac.uk/	2023
17			GEN								√			√		https://ngdc.cncb.ac.cn/gen/	2023
18			TCGA							√	√	√				https://www.cancer.gov/	2023
19			GeneCards							√	√	√				https://www.genecards.org/	2024
20			BiGG							√			√			http://bigg.ucsd.edu/	2023
21			NCBI							√	√	√				https://www.ncbi.nlm.nih.gov/	2024
22			Human Protein Atlas								√	√				https://www.proteinatlas.org/	2023
23			CPTAC							√		√				https://proteomics.cancer.gov/programs/cptac	2023
																	
1			TOXRIC		√		√	√			√				√	https://toxric.bioinforai.tech/	2022
2	**Benchmark**		TDC												√	https://tdcommons.ai/	2023
3			MoleculeNet												√	https://moleculenet.org/	2017
4			MolData												√	https://github.com/LumosBio/MolData	2022
																	
1	**Web server and tools**	**Chemical Toxicity Endpoints**	VenomPred2	Multiple toxicity predictions	√											http://www.mmvsl.it/wp/venompred2/	2023
2			toxCSM	Small molecule toxicity prediction	√					√						https://biosig.lab.uq.edu.au/toxcsm/	2023
3			SApredictor	Structural alert‐based expert system for drug toxicity prediction												https://www.sapredictor.cn/	2022
4			ProTox	Toxic substances of natural or artificial origin	√											http://bioinformatics.charite.de/supertoxic	2015
5			IUCLID	Managing chemical information using a common format	√											https://iuclid6.echa.europa.eu/home	2024
6			cardioToxCSM	Cardiotoxicity prediction	√											https://biosig.lab.uq.edu.au/cardiotoxcsm/	2022
7			MRA Toolbox	Mixture risk assessment	√											https://www.mratoolbox.org/	2023
8		**Protein and Peptide Toxicity**	ToxinPred2	Predicting toxic and non‐toxic proteins	√		√									https://webs.iiitd.edu.in/raghava/toxinpred2	2022
9			CSM‐Toxin	Protein toxicity prediction	√											https://biosig.lab.uq.edu.au/csm_toxin/	2021
10			ToxIBTL	Prediction of peptide toxicity	√											https://server.wei‐group.net/ToxIBTL/Server.html	2022
11		**ADMET**	ADMETboost	ADMET prediction	√											https://ai‐druglab.smu.edu/admet	2022
12			ADMETlab 3.0	ADMET prediction	√											https://admetlab3.scbdd.com/	2023

### 3.1 Toxicology Databases

Toxicology databases collect information on various or specific toxicities of drugs and serve as labeled data for ML‐based toxicity prediction studies.^[^ ^56^ ^]^ We categorize them into four subgroups: universal chemical toxicity databases, organ‐specific databases, carcinogenicity databases, and other toxicity databases. Each subgroup offers different advantages for ML applications in toxicology prediction.

#### 3.1.1 Universal Chemical Toxicity Databases

Universal chemical toxicity databases provide comprehensive toxicity data for a wide range of chemical substances, supporting ML and computational methods in toxicity prediction. Among them, TOXRIC provides ML‐ready datasets, diverse feature data, and benchmark results, making it suitable for rapid model development and testing. Tox21, ToxCast, CompTox Dashboard, and DSSTox are developed by the U.S. Environmental Protection Agency (EPA)’s National Center for Computational Toxicology (NCCT), which provide comprehensive in vitro bioassay data and other chemical information of compounds. EChemPortal is known for its wide chemical records and endpoint coverage. The ToxRefDB provides extensive experimental data coverage and dose‐response models, while ICE is noted for its free access, computational tools, and multi‐endpoint dataset integration. T3DB provides toxin data.

##### TOXRIC

TOXRIC is a comprehensive and accessible online database designed for ML applications.^[^ ^55^ ^]^ It contains data on 113372 chemicals, 13 toxicity categories, and 1474 toxicity endpoints. TOXRIC provides researchers with ML‐ready datasets and benchmarking tools, enabling them to efficiently build predictive models for various toxicity endpoints. TOXRIC 2.0 is currently being updated.

##### Tox21 and ToxCast

Tox21 and ToxCast are both large‐scale, high‐throughput screening databases. Tox21 contains over 10000 chemicals, with data on nuclear receptors and stress response pathways, producing over 150 million data points. ToxCast includes ≈10000 chemicals and 20 assay sources, covering a broad range of biological targets such as mitochondrial toxicity and nuclear receptor signaling.^[^ ^57^ ^]^

##### CompTox Dashboard

The CompTox Chemicals Dashboard houses data on over 1.2 million chemicals.^[^ ^58^ ^]^ It provides information on chemical structures, environmental behavior, and biological activities, supporting batch searches and real‐time predictions of physicochemical properties and toxicological outcomes.

##### DSSTox

DSSTox is a database that underpins Tox21 and ToxCast. Updated in 2024, DSSTox contains over 1 million substances, linking chemical structures to toxicity data.^[^ ^59^ ^]^

##### eChemPortal

eChemPortal has records for 26700 substances and more than 1.3 million chemical property records. Toxicity endpoints in eChemPortal include acute toxicity, chronic toxicity, ecotoxicity, genotoxicity, reproductive toxicity, and carcinogenicity.

##### ToxRefDB

ToxRefDB compiles in vivo toxicity data from over 5900 guideline‐based studies.^[^ ^60^ ^]^ This includes dose‐response models for subacute, chronic, developmental, and reproductive toxicity.

##### ICE

ICE provides free access to in vivo and in vitro test data, alongside computational tools for toxicity prediction.^[^ ^61^ ^]^ ICE integrates toxicity endpoints of acute oral, dermal, inhalation toxicity, cancer, developmental, and reproductive toxicity.^[^ ^62^ ^]^

##### T3DB

T3DB includes 3678 toxins covering pollutants, pesticides, pharmaceuticals, and food, linking to 2073 toxin target records.^[^ ^63^ ^]^ Each ToxCard entry contains chemical properties, identifiers, indicators of toxicity, molecular and cellular interactions, and medical details.

#### 3.1.2 Organ‐Specific Databases

Organ‐specific databases are those focused on the collection of toxicity data specific to the organs or systems. Among them, toxicity data of ToxBank contain multiple organs, while LTKB and LiverTox focus on drug‐induced liver injury.

##### ToxBank

ToxBank contains toxicity, structure, target, and toxicogenomic data for selected chemicals.^[^ ^64^ ^]^ The chemicals are selected by the Gold Chemical Working Group criteria, with a small quantity containing 20 hepatotoxic, 7 cardiotoxic, and 2 nephrotoxic chemicals.

##### Liver Toxicity Database

LTKB and LiverTox both focus on DILI. LTKB detailed drug‐related information, including DILI mechanism, drug metabolism, histopathology, therapeutic use, target, side effects, biomarkers, and a benchmark dataset (LTKB‐BD) of potential drugs linked to human DILI.^[^ ^65^ ^]^ LiverTox provides hepatotoxicity data about prescription, over‐the‐counter drugs, herbs, and supplements.^[^ ^66^ ^]^

#### 3.1.3 Carcinogenicity Databases

Carcinogenicity databases collect data related to chemicals’ carcinogenicity. CCRIS offers multidimensional carcinogenicity information. GENE‐TOX focuses on genotoxicity.

##### CCRIS

The CCRIS contains 9562 chemicals and focuses on carcinogenicity, mutagenicity, and tumor‐promotion data.^[^ ^67^ ^]^ CCRIS provides detailed data on chemicals, including industrial chemicals and environmental agents.

##### GENE‐TOX

GENE‐TOX provides information on the carcinogenicity associated with genotoxic chemicals, focusing on mutagenicity and other genetic damage that may lead to cancer.^[^ ^68^ ^]^ It provides peer‐reviewed genotoxicological test results for more than 3000 chemicals.

#### 3.1.4 Other Toxicity Databases

Other toxicity databases support specific areas of toxicological research and the application of ML in toxicity prediction. RepDose is known for its repeated‐dose toxicity data. ToxDP2 is specialized in predicting the toxicity of dietary polyphenols and food safety research.

##### RepDose

The RepDose database focuses on repeated dose toxicity, containing data on 930 chemicals across 3100 studies.^[^ ^69^ ^]^ It provides values for No Observed Effect Level / Lowest Observed Effect Level (NOEL/LOEL) and other chronic toxicity measures.

##### ToxDP2

The ToxDP2 database fills a gap in food safety research by providing toxicity data on dietary polyphenols.^[^ ^70^ ^]^ It contains data on 415 chemicals.

### 3.2 Chemical Databases

Chemical databases are repositories that collect information on various properties of chemicals and can be used as feature data in ML‐based toxicity prediction studies. They are divided into three subgroups: universal drug databases, chemical‐related target and interaction databases, natural product, and traditional medicine databases.

#### 3.2.1 Universal Drug Databases

Universal drug databases provide detailed information on chemicals. PubChem, DrugBank, ChEMBL, ZINC, and OCHEM provide chemical structures, bioactivities, pharmacological properties, drug targets, and safety data of a wide range of chemicals. ChemSpider offers search tools for accessing chemical data supports QSAR model building and chemical property prediction through large experimental data sets.

##### PubChem

PubChem is an open‐access database from the National Institutes of Health (NIH) focusing on small molecules and macromolecules. It includes extensive data on chemical structures, properties, biological activities, and safety information. The PubChem database currently has over 118 million chemicals, 318 million substances, 296 million bioactive data points, 113 thousand genes, 247 thousand protein targets, and 241 thousand pathways.^[^ ^71^ ^]^

##### DrugBank

DrugBank is a comprehensive resource for drug research, offering detailed information on chemical, pharmacological, and pharmaceutical data for over 16600 drugs.^[^ ^72^ ^]^

##### ChEMBL

ChEMBL collects a large amount of biological activity data, including in vitro, in vivo, and clinical trial data.^[^ ^73^ ^]^ It aims to normalize biological activity into standardized formats.

##### ZINC

ZINC encompasses over 230 million purchasable stock chemicals, all stored in directly dockable 3D formats.^[^ ^74^ ^]^ Additionally, ZINC contains over 750 million purchasable chemicals, with similar molecules searchable within a minute. Molecular data is provided in four formats: isomeric SMILES, mol2, SDF, and flexibase.

##### ChemSpider

ChemSpider covers 129 million chemical structures from 278 data sources, providing structure and substructure searches, as well as validation and organization of synonyms, enhancing the accuracy of text searches.

##### OCHEM

OCHEM database currently contains 4064457 records of 695 attributes (with at least 50 records) collected from 20932 sources.^[^ ^75^ ^]^ Through OCHEM, users can establish QSAR models to predict chemical properties or screen chemical libraries based on structure alerts for endpoints such as mutagenicity, skin sensitization, and aquatic toxicity.

#### 3.2.2 Chemical‐Related Target and Interaction Databases

Chemical‐related target and interaction databases focus on the interactions of chemical substances with biological targets, adverse drug effects, drug interactions, and toxicity‐related biomarkers. TTD provides a wide range of drug‐related targets, diseases, etc. nSIDES integrates adverse reaction data for adults and children. SIDER offers drug‐adverse reaction pair data. TheMarker covers biomarker data.

##### TTD

TTD is dedicated to the collection and curation of drug targets, their associations with diseases, biological functionalities, structural information, as well as drug‐target interactions.^[^ ^76^ ^]^

##### nSIDES

The nSIDES database collects drug side effects and drug interaction information, including specific databases for adult and pediatric adverse reactions (OnSIDES, KidSIDES, OffSIDES, TwoSIDES).

##### SIDER

SIDER focuses on ADRs from marketed drugs, providing data on drug‐ADR pairs, drug indications, and classification to assist in toxicity predictions.^[^ ^77^ ^]^ SIDER encompasses data on 140 drugs, 5880 ADRs, and 140064 drug‐ADR pairs.

##### TheMarker

TheMarker database focuses on biomarkers associated with toxicological outcomes.^[^ ^78^ ^]^ This database can be applied in the identification of early biomarkers for drug‐induced toxicity. It complements other resources like DrugMatrix and TOXRIC in providing a molecular‐level understanding of toxicology.

#### 3.2.3 Natural Product and Traditional Medicine Databases

Natural product and traditional medicine databases gather chemical components, bioactivities, pharmacological effects, and clinical applications of natural products and traditional medicines. Indian Medicinal Plants, Phytochemistry And Therapeutics 2.0 (IMPPAT 2.0) provides chemical and therapeutic information for Indian medicinal plants. The Natural Product Activity and Species Source Database (NPASS) aggregates data on natural product chemicals and bioactivities. Database of Blood‐Absorbed Components and Metabolites of Traditional Chinese Medicine (DCABM‐TCM) offers data on traditional Chinese medicine components and metabolites.

##### IMPPAT

IMPPAT 2.0 comprises data on 4010 Indian medicinal plants, 17967 plant chemical chemicals, and 1095 therapeutic uses.^[^ ^79^ ^]^ The database provides chemical structures, physicochemical properties, drug similarity, and ADMET properties.

##### NPASS

NPASS 2.0 includes 96481 distinct natural chemicals derived from 32287 species, accompanied by 958866 activity entries targeting 7753 different proteins.^[^ ^80^ ^]^ Many drugs are derived directly or indirectly from natural products.^[^ ^81^ ^]^ NPASS provides ML with data characterizing natural products, facilitating their toxicity prediction, and evaluating whether they can become drugs.^[^ ^82^ ^]^

##### DCABM‐TCM

DCABM‐TCM is the collection of blood components of Chinese herbal formulas and individual herbs.^[^ ^83^ ^]^ Core data includes 1816 blood components derived from 194 Chinese herbs and 192 formulas, manually curated from literature.

### 3.3 Omics Databases

With the rapid development of sequencing technology, an increasing amount of omics data has been accumulated. scRNA‐seq can also be used to assess the interaction between chemicals and gene expression.^[^ ^84^ ^]^ Omics databases collect data across various omics layers, which can be used as feature data for drugs, proteins, and cells in ML‐based toxicity prediction studies.^[^ ^85^ ^]^ These databases are categorized into four types: genomic and transcriptome databases, proteomics databases, metabolomics databases, and multi‐omics databases.

#### 3.3.1 Genomic and Transcriptome Database

Genomic and Transcriptomic Databases collect genome and transcriptome data. Among them, Open TG‐GATEs and DrugMatrix provide multidimensional transcriptome data, PharmGKB focuses on pharmacogenomics and variability in drug responses, ToxicoDB offers in vitro gene expression data, and CTD integrates data on chemicals, genes, diseases, and exposure events. Gene Expression Omnibus (GEO) provides a vast amount of gene expression data, Kyoto Encyclopedia of Genes and Genomes (KEGG) helps to understand the interactions between drugs and biological networks through structured gene and metabolic pathway information, and Catalogue of Somatic Mutations in Cancer (COSMIC) specializes in cancer somatic mutation data, aiding in the prediction of drug carcinogenicity.

##### Open TG‐GATEs

Open TG‐GATEs is a toxicogenomics database containing data on 170 chemicals (mainly drugs) that have been exposed in rats (in vivo) and in primary rat and human hepatocytes (in vitro).^[^ ^86^ ^]^ The database provides gene expression data, biochemical, hematological, and histopathological findings, as well as pathology imaging from in vivo studies, and cytotoxicity data from in vitro studies.

##### PharmGKB

PharmGKB offers various pharmacogenomics (PGx) information, which aims to help people understand how genetic variations lead to differences in drug responses.^[^ ^87^ ^]^ Currently, the database contains 201 clinical guideline annotations, 1059 drug label annotations, 457 FDA drug label annotations, and 41 curated pathways.

##### ToxicoDB

ToxicoDB integrates data from in vitro toxicogenomics studies, including gene expression profiles of primary human and rat hepatocytes treated with 231 potential toxicants.

##### DrugMatrix

DrugMatrix is a comprehensive toxicogenomics database developed by the National Toxicology Program (NTP) and managed by the National Institute of Environmental Health Sciences (NIEHS).^[^ ^88^ ^]^

##### CTD

CTD curates and interrelates chemicals, genes, phenotypes, anatomy, diseases, taxa, and exposure information from published literature, coordinating cross‐species heterogenous data on chemical exposure and its biological effects.^[^ ^89^ ^]^ CTD presently contains data on 17100 chemicals, 54300 genes, 6100 phenotypes, 7270 diseases, and 202000 exposure records.

##### GEO

The role of GEO in ML models lies in its massive collection of gene expression data, containing both microarray and RNA‐seq data.^[^ ^90^ ^]^ This data can be used to train models that predict how different chemicals or drugs affect gene expression, offering insight into potential toxic effects at the molecular level.

##### KEGG

KEGG is an encyclopedia of genes and genomes, which offers structured information on metabolic pathways, molecular interactions, and gene functions.^[^ ^91^ ^]^ ML models can use this information to map how drugs interact with biological networks. By integrating KEGG pathways as prior knowledge into ML models, researchers can better interpret predictions and understand the underlying mechanisms of drug toxicity, including off‐target effects and drug‐drug interactions.

##### COSMIC

COSMIC is pivotal for ML models focusing on carcinogenicity prediction.^[^ ^92^ ^]^ By incorporating somatic mutation data from cancer samples, ML algorithms can assess how specific mutations may interact with drugs, potentially leading to cancerous transformations.

#### 3.3.2 Proteomics Database

Proteomics databases provide rich protein sequences, functional annotations, and interaction information, offering key molecular‐level data for ML models. These databases effectively support drug toxicity prediction, particularly in analyzing drug‐protein interactions and toxicity mechanisms. InterPro offers detailed functional protein annotations and information on families and domains, facilitating cross‐domain ML and TL. UniProt, with its extensive protein sequence and annotation data, supports toxicity prediction of drug‐protein interactions, especially in hepatotoxicity and nephrotoxicity.

##### InterPro

InterPro's functional protein annotations provide valuable insights into protein families, structural domains, and important protein sites.^[^ ^93^ ^]^ ML models benefit from the integration of this detailed protein feature data to predict how drugs interact with proteins, which is key to predicting toxicity at the molecular level.

##### UniProt

UniProt's vast collection of protein sequences and annotations enables ML models to predict toxic reactions caused by drug‐protein interactions.^[^ ^94^ ^]^

#### 3.3.3 Metabolomics Database

Metabolomics databases supply metabolite data and related metabolic pathway information, aiding in drug metabolism product toxicity prediction and identifying potential toxic mechanisms. METLIN provides precise mass spectrometry data and metabolite structure information, and HMDB integrates chemical, clinical, and molecular biology data. The LIPID Metabolites and Pathways Strategy (LIPID MAPS) focuses on lipid metabolites and pathways, while MetaCyc/BioCyc offers cross‐species metabolic pathway data.

##### METLIN

METLIN's tandem mass spectrometry data for metabolites can be integrated into ML models for predicting the toxic effects of drug metabolism.^[^ ^95^ ^]^ ML models trained on METLIN data can identify metabolic byproducts of drug biotransformation that may cause toxicity.^[^ ^96^ ^]^

##### HMDB

HMDB supports ML models by providing chemical, clinical, and molecular biology data on small molecule metabolites.^[^ ^85^ ^]^ This comprehensive dataset allows models to learn the associations between specific metabolites and toxic responses in the human body.

##### MssBank

MassBank's repository of mass spectral data plays a crucial role in ML models focused on exposomics and drug metabolism. The spectral matching feature of MassBank aids in validating ML model predictions about metabolic profiles related to toxicity in vivo.

##### LIPID MAPS

LIPID MAPS provides lipidomics data.^[^ ^97^ ^]^ ML models can use its lipid structure data to predict drug‐lipid interactions.

##### MetaCyc/BioCyc

MetaCyc/BioCyc provide metabolic pathways across species.^[^ ^98^ ^]^ Pathway‐based ML models can use MetaCyc's detailed pathway descriptions to simulate how drugs alter metabolic processes, helping predict off‐target toxic effects.^[^ ^99^ ^]^

#### 3.3.4 Multi‐Omics Database

Multi‐omics databases integrate genomic, transcriptomic, proteomic, and metabolomic data, providing a comprehensive biological perspective for ML models. The European Bioinformatics Institute (EMBL‐EBI) offers extensive multi‐omics data support, and Gene Expression Nebulas (GEN) focuses on transcriptomics and single‐cell data. The multi‐omics data of The Cancer Genome Atlas (TCGA) highlights cancer‐related multi‐omics data, GeneCards integrates genetically relevant multi‐omics data, and BiGG specializes in genomic and metabolic data. The National Center for Biotechnology Information (NCBI) excels in providing genomic, transcriptomic, proteomic, variant data, and gene function annotations. The Human Protein Atlas (HPA) provides comprehensive, open‐access data across multiple omics technologies. The proteomics data of the Clinical Proteomic Tumor Analysis Consortium (CPTAC) provides large‐scale proteome and genome analysis data for cancer.

##### EMBL‐EBI

EMBL‐EBI offers integrated multi‐omics datasets, including genomics, transcriptomics, proteomics, and metabolomics, leading to more robust predictions of drug toxicity and mechanisms of action.^[^ ^100^ ^]^

##### GEN

GEN provides large‐scale transcriptomic data, including single‐cell RNA sequencing datasets.^[^ ^101^ ^]^

##### TCGA

By combining TCGA's genomic, transcriptomic, and proteomic data, models can predict how specific cancer treatments may cause toxic side effects, aiding in personalized medicine approaches.^[^ ^102^ ^]^

##### GeneCards

GeneCards offers an integrated view of genes and their associated omics data, which supports ML models in toxicity prediction by linking genetic variations with drug responses.^[^ ^103^ ^]^ The combination of genomic, proteomic, and transcriptomic data in GeneCards facilitates the development of personalized toxicity prediction models, where individual genetic backgrounds are considered when evaluating a drug's safety profile.

##### BiGG

The BiGG Models Knowledge Base provides genome‐scale metabolic networks.^[^ ^104^ ^]^ These models help identify metabolic bottlenecks or imbalances that could lead to toxicity. BiGG offers benchmark content to assess and validate the quality of genome‐scale metabolic models. By offering a unified system of metabolite and reaction identifiers, BiGG facilitates the integration of metabolic models into broader ML pipelines for toxicity prediction.

##### NCBI

NCBI offers a wide range of bioinformatics resources, including genomic and molecular data.^[^ ^105^ ^]^ ML models can leverage these resources to develop comprehensive predictive systems that consider multiple biological factors, from gene sequences to protein interactions. NCBI's data also allows models to integrate genetic information with chemical toxicity data, improving predictions of how genetic variations influence drug responses.

##### Human Protein Atlas

HPA provides detailed protein expression data across tissues, enabling ML models to predict tissue‐specific toxic effects of drugs.^[^ ^106^ ^]^ By integrating proteomics data with transcriptomics and genomics, models can identify how drugs interact with proteins in specific tissues, helping to predict adverse reactions such as cardiotoxicity or hepatotoxicity.

##### CPTAC

CPTAC supports ML models focused on predicting cancer‐related drug toxicity.^[^ ^107^ ^]^ By combining protein‐level data with genomic and transcriptomic information, models can identify the proteomic changes that accompany drug resistance or toxicity in cancer therapies. CPTAC's rich dataset enables models to explore post‐translational modifications and their impact on drug efficacy and safety.

These databases provide vast, diverse datasets that capture various biological layers, from gene sequences to metabolic pathways, enabling models to make more accurate and interpretable predictions. By leveraging these resources, ML models can simulate complex biological processes, predict adverse drug reactions, and support the development of safer and more effective drugs.

### 3.4 ML Benchmark Databases

ML Benchmark databases provide benchmark results for ML models. TOXRIC offers standardized datasets, various types of benchmarks, and molecular representations, providing strong benchmark results. TDC provides standardized datasets and leaderboards to ensure model reliability. MoleculeNet covers a wide range of molecular properties and supports evaluations with DL, while MolData aids drug repurposing and toxicity prediction through multi‐task learning and cross‐disease category support.

#### 3.4.1 TOXRIC

In addition to toxicity data, TOXRIC provides two types of benchmarks and visualizations of molecular representations for all toxicity endpoint subsets.^[^ ^55^ ^]^ For the benchmarks on feature types, 36 features are tested. For the benchmarks on different algorithms, four typical toxicity prediction algorithms with the same input are tested.

#### 3.4.2 TDC

TDC includes task‐specific toxicity data that can be used for benchmarking ML models.^[^ ^108^ ^]^ By offering standardized datasets and leaderboards, TDC fosters reproducibility and limits erroneous conclusions.

#### 3.4.3 MoleculeNet

MoleculeNet is a comprehensive benchmark for molecular properties, including toxicity.^[^ ^109^ ^]^ MoleculeNet's benchmark results (AUC‐ROC, RMSE, etc.) help assess the effectiveness of these models in predicting toxic molecular behaviors.

#### 3.4.4 MolData

MolData provides a collection of biological assay data across diseases and drug targets, including more than 170 million drug test outcomes.^[^ ^110^ ^]^ Besides, the LTKB and BiGG databases also provide benchmark data, as introduced in the sections on toxicology and multi‐omics.

### 3.5 Web Servers and Tools

Web servers and tools provide rapid visualization, analysis, and quantification of chemical toxicity predictions. We categorize tools used for toxicity prediction into three categories: chemical toxicity prediction tools, protein and peptide toxicity prediction tools, and ADMET prediction tools.

#### 3.5.1 Chemical Toxicity Prediction Tools

Chemical Toxicity Prediction tools predict various toxicity endpoints for small molecule chemicals. VenomPred provides rapid multi‐endpoint toxicity prediction using ML and consensus strategies. toxCSM uses graph features and molecular descriptors to predict environmental and stress response toxicity. SApredictor optimizes molecular structures to reduce toxicity. ProTox supports predicting multiple toxicity endpoints and toxicological pathway analysis. Internationally Unified Chemical Information Database (IUCLID) offers a unified platform for managing and analyzing chemical toxicity data. CardioToxCSM focuses on cardiotoxicity prediction and MRA Toolbox provides tools based on pattern recognition and risk assessment.

##### VenomPred

VenomPred 2.0 provides toxicity prediction across multiple endpoints, such as cancer risk, liver toxicity, genetic mutations, and skin irritation.^[^ ^111^ ^]^ VenomPred leverages ML models and consensus strategies, allowing users to input chemical structures (via SMILES strings) for rapid toxicity assessment, including identifying toxic groups and structural fragments.

##### toxCSM

toxCSM focuses on predicting the toxicity of small molecules using supervised learning with graph‐based features and molecular descriptors.^[^ ^112^ ^]^ It supports toxicity prediction in various contexts like environmental and stress response toxicities and helps in designing safer drugs, herbicides, and insecticides. However, toxCSM's performance on new or unseen chemicals requires further validation.

##### SApredictor

SApredictor identifies and highlights SAs linked to critical toxicity endpoints.^[^ ^113^ ^]^ This tool visually marks toxic substructures, aiding medicinal chemists in optimizing molecular structures to minimize toxicity.

##### ProTox

ProTox predicts various toxicity endpoints (acute and organ toxicity, molecular initiating events, metabolism, adverse outcome pathways, etc.) using ML models and molecular similarity features.^[^ ^114^ ^]^ It supports predictions for toxicological pathways, aiding in the early identification of toxic chemicals.

##### IUCLID

IUCLID provides a unified platform for managing and analyzing chemical toxicity data, particularly for reproductive and endocrine toxicity endpoints.^[^ ^115^ ^]^ It supports regulators and industries by facilitating data correlation and consistency analyses, helping develop non‐animal alternatives for toxicity testing.^[^ ^116^ ^]^

##### cardioToxCSM

cardioToxCSM specifically targets cardiac toxicity prediction, assessing six categories of heart toxicity outcomes.^[^ ^117^ ^]^ The platform uses graph‐based features and molecular descriptors to predict risks of arrhythmia, heart failure, hERG toxicity, and other cardiac conditions, aiding in early drug screening for cardiotoxicity.

##### MRA Toolbox

MRA Toolbox evaluates the toxicity of chemical mixtures by modeling combined risks using computational techniques.^[^ ^118^ ^]^ It helps predict dose‐response relationships and Effective Concentration 50%/Lethal Concentration 50% (EC50/LC50) values for mixtures, allowing risk assessors to evaluate and mitigate mixture toxicity during product development.

#### 3.5.2 Protein and Peptide Toxicity Prediction Tools

Protein and Peptide Toxicity Prediction tools are platforms utilizing ML and DL to predict protein and peptide toxicity by analyzing protein sequence and structural features, helping minimize toxicity risks in biopharmaceutical development.

##### ToxinPred2

ToxinPred2 predicts protein and peptide toxicity using ML models such as RF and hybrid methods (RF+ Basic Local Alignment Search Tool, BLAST+ ML Ensemble for Regulation of Chemical Interactions, MERCI).^[^ ^119^ ^]^ It analyzes protein sequences to identify toxic proteins, assisting in biological drug development by minimizing potential toxicity risks.

##### CSM‐Toxin

CSM‐Toxin predicts protein toxicity using DL models pre‐trained with ProteinBERT.^[^ ^120^ ^]^ This server focuses on identifying toxic proteins and peptides, enabling users to input protein sequences for toxicity analysis in biological drug development.

##### ToxIBTL

ToxIBTL uses information bottleneck and TL techniques to predict peptide and protein toxicity.^[^ ^121^ ^]^ It extracts essential information from protein sequences to improve feature representation and predict toxicity, supporting safety assessments of peptide drugs.

#### 3.5.3 ADMET Prediction Tools

ADMET prediction tools are used to predict the ADMET properties of drugs. ADMET is a critical factor in drug efficacy and safety. Different platforms use various methods for ADMET prediction.

##### ADMETboost

ADMETboosts focuses on predicting ADMET properties using tree‐based ML models.^[^ ^122^ ^]^ It performs exceptionally well in the TDC ADMET benchmark, helping researchers predict the ADMET properties of new chemicals.

##### ADMETlab

ADMETlab 3.0 evaluates ADMET properties and drug physicochemical characteristics using the multitask Directed Message Passing Neural Network (DMPNN) framework.^[^ ^123^ ^]^ This tool enables rapid and accurate toxicity prediction, including uncertainty estimation for reliable chemical selection.

## 4 Challenges and Opportunities for Future ML in Drug Toxicity Prediction

**Figure 5:**
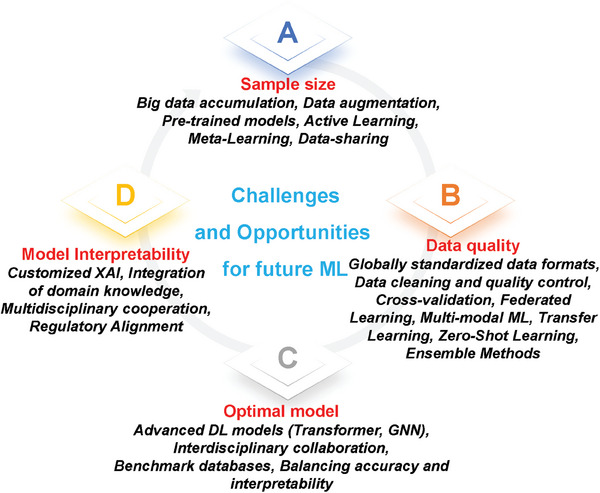
Challenges and Opportunities for Future ML in Drug Toxicity Prediction. Although ML has advanced drug toxicity prediction, it still faces challenges and significant opportunities. We summarize four key challenges, with italicized text explaining how to address them. A) Sample Size: The small size of available datasets limits ML performance. Advances in big data accumulation, data augmentation, pre‐trained models, active learning, and meta‐learning, as well as improved data sharing and integration, can help overcome this challenge. B) Data Quality: Poor data quality hampers model reliability. Data cleaning, quality control, standardized global data formats, federated learning, multimodal ML, transfer learning, zero‐shot learning, and ensemble methods are key strategies to improve data quality. C) Optimal Model: Finding suitable ML models for drug toxicity prediction is difficult. Emerging DL models (Transformers and GNNs), interdisciplinary collaboration, creating benchmark databases, and balancing accuracy with interpretability are essential for selecting the right models. D) Model Interpretability: Limited interpretability complicates ML application. Developing customized interpretable artificial intelligence (XAI) methods for different data types, fostering Multidisciplinary cooperation, integrating domain knowledge, and aligning with regulatory frameworks can address challenges in model applicability, the lack of interpretability metrics, and data complexity.

Despite the numerous advancements in ML‐assisted toxicology, it continues to encounter several challenges and prospects (**Figure** **5**).

### 4.1 Expanding Sample Sizes

Inadequate sample size is the first challenge, leading to suboptimal model training, and affecting prediction accuracy and generalization ability. As mentioned above, there is a shortage of labeling data on clinical toxicity, nephrotoxicity, respiratory toxicity, neurotoxicity, and mitochondrial toxicity. With the progress and widespread adoption of sequencing technologies, multi‐omics data and toxicity data based on drug perturbations are rapidly accumulating.^[^ ^124^ ^]^ Improvements in ML algorithms, such as data augmentation, pre‐trained models, active learning, and meta‐learning, can increase the existing sample size.^[^ ^125^ ^]^ Collaborative data sharing and integration further increase sample size, with platforms like Tox21, ToxCast, and TOXRIC aggregating data from multiple sources, while public engagement and open‐access policies increase dataset growth. These strategies may collectively enhance ML performance and reliability in toxicity prediction.

### 4.2 Improving Data Quality and Integrating Diverse Sources

The scarcity of high‐quality data and challenges in integrating multi‐source datasets limit ML applications. Standardizing data formats using principles like FAIR ensures data interoperability, whole rigorous data cleaning, and cross‐validation pipelines minimize noise and errors. Integrating various data sources for toxicity prediction faces challenges such as data quality, heterogeneity, and sparsity. Techniques such as Federated Learning (FL), multi‐modal ML, TL, ZSL, and ensemble methods help integrate toxicological, chemical, and multi‐omics data to address data scarcity issues. FL enables collaborative training without sharing raw data, addressing privacy concerns that hinder cross‐institution collaboration. Multi‐modal ML combines diverse data types (e.g., genomics, imaging, chemical structure) to improve robustness.^[^ ^126^ ^]^ TL reduces reliance on large datasets by adapting pre‐trained models to new data. For instance, pre‐training models on datasets like Tox21 or PubChem, and fine‐tuning them on specific toxicity types or omics data, improves model generalization and addresses domain‐specific data scarcity. ZSL predicts the toxicity of unseen chemicals by transferring knowledge between related datasets. Synthetic data generation techniques create artificial data to simulate features of unseen categories, allowing models to train on synthetic data with similar statistical properties to real‐world data, thus improving performance on actual data. Synthetic data generation techniques provide an effective means for ZSL to address data scarcity and improve the model's ability to identify unseen categories. Personalized toxicity prediction based on patient‐specific data or using synthetic data generation techniques can help address data scarcity. Combining FL, TL, and ZSL into standardized workflows helps merge multi‐source data into cohesive frameworks. These methods effectively integrate and enhance multi‐source datasets, creating high‐quality unified data for toxicity prediction and advancing ML applications in toxicology.

### 4.3 Optimizing ML Models for Diverse Toxicity Prediction

Predicting diverse toxicities like acute toxicity, carcinogenicity, and organ‐specific toxicity requires specialized models due to their unique mechanisms. Different types of toxicity may require different feature sets or representations. For instance, chemical toxicity might be better represented by molecular descriptors, while biological toxicity may be more effectively captured by gene expression profiles. Liver toxicity, closely linked to metabolic enzyme activity, bile acid cycling, and oxidative stress, can be accurately predicted by integrating liver‐specific multi‐omics data and dynamic response models. For different toxicity categories, appropriate input representations and prediction models must be established separately to achieve further performance improvements.

The emergence of DL models offers opportunities to overcome challenges in predicting different types of toxicity. Compared to traditional models, emerging DL technologies like transformers and GNN can capture more complex nonlinear relationships in the data. Moreover, these models are highly scalable and can handle large datasets, which is particularly beneficial for tasks involving high‐dimensional chemicals and biological data. Collaboration among toxicologists, data scientists, biologists, and clinicians is essential to develop suitable DL workflows that incorporate patient data, such as genetic information and clinical data, enabling personalized predictions and improved treatment outcomes.

Building more benchmark databases is crucial for constructing and validating toxicity prediction models. However, current benchmark databases often lack standardization, hindering reproducibility and cross‐model comparisons. Establishing shared, curated benchmarks like TOXRIC, TDC, and MoleculeNet can improve evaluation consistency. Researchers are encouraged to open‐source their code, hyperparameter details, and data preprocessing steps through repositories such as GitHub or Zenodo, improving the reproducibility of model predictions. Developing standardized benchmarks and reporting guidelines will further ensure reliability and accelerate advancements in toxicity prediction.

ML's accuracy remains suboptimal in many toxicity areas despite strong performance in specific domains. Prioritizing model accuracy is crucial, with interpretability improvements following. In industrial applications, computational efficiency and accuracy are of greater concern. High‐performance models should be developed first, with iterative optimizations to facilitate the widespread adoption of ML‐driven toxicity prediction in real‐world scenarios.

### 4.4 Enhancing Explainability in ML for Drug Toxicity Prediction

In drug toxicity prediction, limited interpretability is a major challenge for ML models. The goal of interpretability is to provide a transparent and traceable prediction process, helping researchers understand the decision‐making mechanisms of ML models. This enhances the model's credibility, usability, and acceptance, which is crucial for clinical decisions and regulatory approvals. Advanced methods like SHAP and LIME can handle various data types but often require customization for complex biological data (e.g., time series, multimodal, and high‐dimensional sparse data).^[^ ^127^ ^]^ The absence of recognized evaluation standards makes it difficult to quantify or assess interpretability methods. Current evaluation methods are limited to simple qualitative analysis or restricted quantitative metrics, failing to meet practical application needs. The diversity and complexity of biomedical data (e.g., genomic, omics, and clinical data) further complicate the application of XAI.^[^ ^128^ ^]^

With the advancement of XAI technology, customized methods for different data types are emerging, such as probing, perturbation, and surrogate models.^[^ ^127^ ^]^ The development of supporting tools and libraries (such as DeepLIFT and GNNExplainer) significantly improves the application and integration of XAI methods.^[^ ^129^ ^]^ Furthermore, multidisciplinary collaboration and integration of domain knowledge are opening new directions for XAI in drug toxicity prediction. This includes using knowledge graphs to validate whether explanations align with biological logic and quantifying knowledge consistency.^[^ ^130^ ^]^

By overcoming these challenges and aligning with regulatory frameworks, AI's potential in regulatory toxicology is being realized. ML models must meet transparency and reproducibility standards and gain regulatory approval through standardized protocols. AI can drive the development of reliable in vitro and computational models, reducing reliance on animal testing,^[^ ^131^ ^]^ and improving toxicity prediction performance and the understanding of toxicological mechanisms by integrating multi‐omics and chemical data. Automated and interpretable models can help regulatory agencies prioritize chemicals based on quantitative predictions, guiding further testing. These advancements make ML a key foundation for modern regulatory toxicology practices.

## 5 Conclusion

In this review, we highlighted the growing role of ML in addressing challenges associated with drug toxicity prediction. By categorizing 10 types of drug‐induced toxicities and analyzing their characteristics and predictive models, we demonstrated how ML can bridge prediction accuracy and mechanistic understanding. Additionally, our comprehensive summary of 55 databases and 12 tools offers researchers valuable resources to explore toxicity across diverse domains, facilitating data‐driven insights and informed decision‐making. These efforts emphasize the potential of ML to overcome the limitations of traditional toxicity assessment methods, including high costs, ethical concerns, and cross‐species variability.

Despite these advancements, significant challenges remain, such as data scarcity, model interpretability, and multi‐source data integration. Addressing these issues requires continued development of robust and interpretable ML models, supported by standardized evaluation metrics and cross‐disciplinary collaboration. By turning challenges into opportunities, ML can further drive innovation in toxicology, reducing reliance on animal experiments and paving the way for safer and more effective drug discovery.

## Conflict of Interest

The authors declare no conflict of interest.

## Supporting information



Supporting Information
