# Supplementary material for: Machine Learning‐Enabled Drug‐Induced Toxicity Prediction
Source: Adv Sci (Weinh). 2025 Feb 3;12(16):2413405. doi: 10.1002/advs.202413405 (PMC12021114; doi:10.1002/advs.202413405)
Supplement: Supplementary file 1 — Supporting Information [file ADVS-12-2413405-s001.docx]

Supporting Information

Machine Learning-enabled Drug-induced Toxicity Prediction

Changsen Bai^#^, Lianlian Wu^#^, Ruijiang Li, Song He^*^, Xiaochen Bo^*^

**Table S1 Different ML Models Prediction Performance Across Toxicity Types**

| Algorithms | Toxicity Category | | | | | | | | | |
| --- | --- | --- | --- | --- | --- | --- | --- | --- | --- | --- |
|  | Acute toxicity | Clinical toxicity | Carcinogenic | Hepatotoxicity | Cardiotoxicity | Nephrotoxicity | Respiratory toxicity | Neurotoxicity | Hematotoxicity | Mitochondrial Toxicity |
| LR |  |  | ○ | ○ | ○ | ○ | ○ | ○ | ○ |  |
| DT |  |  |  | ○ | ○ |  |  | ○ | ○ |  |
| SVR | **☆** |  |  |  |  |  |  |  |  |  |
| SVM |  |  | ○ | **☆** | ○ | ○ | **☆** | **☆** | ○ |  |
| RVM |  |  |  |  |  |  | ○ |  |  |  |
| BB |  |  |  | ○ |  |  |  |  |  |  |
| RF | **☆** |  | ○ | **☆** | **☆** | ○ | ○ | **☆** | ○ | **☆** |
| RRF |  |  |  |  |  |  | ○ |  |  |  |
| XGB | ○ |  | ○ | **☆** | ○ | ○ | **☆** |  | **☆** | ○ |
| KNN |  |  | ○ |  | ○ |  | ○ | ○ |  |  |
| ADB |  |  | ○ |  |  |  |  |  |  |  |
| SGD |  |  | ○ |  |  |  |  |  |  |  |
| BN |  |  |  | **☆** |  |  |  |  |  |  |
| GBM |  |  | **☆** |  |  |  |  |  |  |  |
| LGBM | ○ |  |  | ○ |  |  |  |  | ○ | ○ |
| ERT |  |  |  |  |  | ○ |  |  |  |  |
| LDA |  |  |  |  | ○ |  | ○ |  |  |  |
| NB |  |  |  |  | ○ |  | **☆** | ○ |  |  |
| NN |  |  |  | ○ |  |  |  |  |  |  |
| ABDT |  |  |  |  |  |  | ○ |  |  |  |
| MLP |  |  | ○ |  |  |  | ○ |  |  |  |
| GBDT |  |  |  | ○ |  |  |  |  | ○ |  |
| CatBoost |  |  |  |  |  |  |  |  |  | **☆** |
|  |  |  |  |  |  |  |  |  |  |  |
| DNN | ○ |  | ○ | **☆** | ○ | **☆** |  |  |  |  |
| GCN | ○ |  | ○ |  | **☆** |  | ○ |  | ○ |  |
| GAN | ○ |  | ○ | **☆** |  |  |  |  |  |  |
| GAT | **☆** |  |  | **☆** | **☆** |  | ○ |  |  |  |
| Attentive FP | ○ |  | ○ |  |  |  |  |  | **☆** |  |
| CNN |  |  |  | ○ |  |  | ○ | **☆** |  |  |
| GraphSAGE | ○ |  |  |  |  |  |  |  |  |  |
| GIN | ○ |  |  |  |  |  |  |  |  |  |
| MolFPG | **☆** |  |  |  |  |  |  |  |  |  |
| MPNN | ○ |  |  |  | ○ |  | **☆** |  | ○ |  |
| PAGTN | ○ |  |  |  |  |  |  |  |  |  |
| SSM |  |  |  | ○ |  |  |  |  |  |  |
| Capsule network+GAT |  |  | **☆** |  |  |  |  |  |  |  |
| GNN |  |  | ○ | ○ |  |  |  |  |  |  |
| GNN+MLP |  |  | **☆** |  |  |  |  |  |  |  |
| SSM |  |  |  |  |  |  |  |  |  |  |
| Mixed Learning |  |  |  | ○ |  |  |  |  |  |  |
| UGRNN |  |  |  | ○ |  |  |  |  |  |  |
| Ensemble Model |  |  |  | ○ |  |  |  |  |  |  |
| GRU |  |  |  |  | ○ |  |  |  |  |  |
| NLP |  |  |  |  | **☆** |  |  |  |  |  |
| LSTM |  |  |  |  | ○ | **☆** |  |  |  |  |
| Smiles2vec |  |  |  |  | ○ |  |  |  |  |  |
| hERG-Att |  |  |  |  | ○ |  |  |  |  |  |
| RNN |  |  |  |  |  | ○ |  |  |  |  |
| GCM |  |  | ○ |  |  |  | ○ |  |  |  |
| STDNN |  | ○ |  |  |  |  |  |  |  |  |
| MTDNN |  | **☆** |  |  |  |  |  |  |  |  |
| TL |  | ○ |  |  |  |  |  |  |  |  |

○ indicate that this ML algorithm was used by a specific study for that toxicity prediction, and ☆ indicate that this ML algorithm was used by a specific study for that toxicity prediction and achieved good performance.
